# Engineered Biochar for Gaseous Air Pollutant Control: Modification Strategies, Synergistic Adsorption Mechanisms, and Comparative Evaluation with Conventional Adsorbents

**DOI:** 10.3390/molecules31203780

**Published:** 2026-10-21

**Authors:** Hongchi Chen, Lei Zhang, Xu Yang, Yujie Cheng, Jiao Ma, Shuai Guo, Zhuozhi Wang, Boxiong Shen

**Affiliations:** 1 School of Chemical Engineering, Hebei University of Technology, Tianjin 300401, China; 2 School of Energy and Power Engineering, Northeast Electric Power University, Jilin 132012, China

**Keywords:** engineered biochar, air pollutants, adsorption mechanisms, modification strategies, structural feature

## Abstract

Biochar is a carbon-rich material derived from the pyrolysis of biomass. It is drawing more attention for air pollutant control because of its low cost, abundant feedstocks, and environmental sustainability. This review examines the application of engineered biochar to control gaseous air pollutants. Meanwhile, the preparation and modification methods for engineered biochar, including pyrolysis, activation, acid–base treatments, amine functionalization, and metal loading, are also systematically summarized. These approaches are used for tailoring pore structural features and the distribution of surface functional groups. Moreover, the review clarifies the synergistic mechanisms of physisorption–chemisorption for CO_2_, SO_2_, NO*x*, and VOCs, emphasizing the cooperative regulation resulting from nitrogen-containing functional groups and metal sites. For example, nitrogen-modified biochar increased SO_2_ adsorption capacity from 78.9 mg/g for pristine biochar to 135 mg/g under mixed gas conditions, while Na_2_CO_3_-activated cattail-derived biochar achieved an n-hexane dynamic adsorption capacity of 1.00 g/g. Subsequently, this study comprehensively compared engineered biochar with conventional adsorbents, including activated carbon, zeolites, carbon molecular sieves, and metal–organic frameworks, considering gas-phase pollutant removal efficiency, removal cost, and operational difficulty of supporting equipment. Although biochar is slightly inferior to high-end commercial activated carbon in terms of adsorption performance, it can be modified to achieve a level comparable to that of activated carbon. As the raw materials for biochar are derived from waste, the cost is virtually zero, and the production cost is approximately 50–80% lower than that of commercial activated carbon. These results demonstrate the potential of engineered biochar as a low-cost, sustainable alternative for controlling industrial gaseous pollutants.

## 1. Introduction

Air pollution has become a critical global environmental challenge, posing serious threats to ecosystems and human health. The primary sources of emissions include industrial activities, emissions from livestock and poultry farming, the burning of fossil fuels, and vehicle exhaust, which release a variety of pollutants, including greenhouse gases, acidifying gases, and volatile organic compounds (VOCs) [1]. Greenhouse gas (GHG) emissions from the livestock sector account for approximately 14.5% of total global emissions. VOCs are highly reactive and can form secondary pollutants, such as ozone. Some VOCs also display significant toxicity, including carcinogenic and teratogenic effects [2]. Greenhouse gases and acidic gases also contribute significantly to climate change and acid rain [3,4]. At present, a wide range of air pollution control technologies have been developed and widely applied to tackle air pollution, including catalytic combustion and absorption [5,6,7,8]. Whilst each technology has its own advantages, it also has inherent limitations that restrict its practical application under various operating conditions. Absorption technology is suitable for the continuous treatment of high-flow-rate gas streams and is relatively simple to operate. However, its treatment efficiency is fundamentally dependent on the solubility of the pollutants in the absorbent. Hydrophobic VOCs generally exhibit poor mass transfer in water-based absorbents, requiring the addition of organic solvents or surfactants to improve removal efficiency; this not only increases operating costs but also entails additional costs associated with solvent recovery and secondary wastewater treatment [9]. Consequently, the absorption method is less economical for low-concentration or poorly soluble VOCs. Catalytic combustion is highly effective for the complete oxidation of VOCs at relatively low temperatures and is therefore widely used in industrial emission control. However, its performance depends to a large extent on the composition of the catalyst and the operating conditions, whilst catalyst deactivation, chlorine poisoning and the formation of toxic chlorinated by-products remain significant challenges. For example, in the γ-Al_2_O_3_-supported vanadium-based catalyst (10V-Al) system, under low-temperature conditions with a chlorobenzene conversion rate of 10%, the total yield of PCDD/Fs can reach 25.60 mmol/mol CB [10]. Consequently, although catalytic oxidation offers high removal efficiency, its environmental sustainability is compromised when treating chlorinated VOCs. Compared with catalytic oxidation and absorption, adsorption technology has attracted increasing attention due to its ability to efficiently capture pollutants under ambient temperature and pressure conditions, without requiring high energy input or generating significant secondary emissions. Conventional adsorbents, including activated carbon, carbon nanotubes, carbon molecular sieves, zeolites, and metal–organic frameworks (MOFs), typically exhibit excellent adsorption capacities [11,12,13]. However, their widespread industrial application remains constrained by high material costs, the need for regeneration, and, in some cases, limited stability under conditions of humid or chemically complex flue gas. These limitations highlight the need to develop low-cost, highly efficient, and environmentally friendly adsorbents that combine high adsorption performance, good regenerative capacity, and long-term operational stability.

In contrast, biochar has emerged as a highly promising alternative due to its low cost, renewability, and adjustable physicochemical properties. Owing to its high porosity, large specific surface area, and abundance of functional groups, biochar demonstrates significant potential for the adsorption of gaseous pollutants, including SO_2_, NO*x*, CO_2_, dioxins, and VOCs [14,15]. Biochar is a carbon-rich material produced by thermochemical conversion of biomass, which is attracting significant attention as an effective material for pollutant remediation [16]. Its feedstocks include agricultural residues, forestry wastes, municipal sludge, and animal manure, which are abundant and low-cost, favoring large-scale production and sustainable environmental applications [17,18,19]. Raw biochar produced by biomass pyrolysis typically has a lower specific surface area and porosity, resulting in poorer structural and adsorption properties. Engineered biochar generally refers to biochar that has undergone modification, functionalization, or composite treatment. Compared with raw biochar, engineered biochar exhibits higher adsorption performance and selectivity [20]. Raw biochar’s well-developed porous structure, high specific surface area, abundant surface functional groups, and chemical stability make it a promising adsorbent. Biochar applications have been extensively studied in several environmental fields [21,22]. These cases demonstrate that biochar possesses adsorption properties, while factors such as the type of feedstock and pyrolysis conditions determine its characteristics, including its pore structure, specific surface area, and the distribution of functional groups. All of these characteristics directly influence its adsorption capacity.

Recent studies have systematically investigated the relationships among biochar preparation conditions, physicochemical properties, and adsorption performance. For example, Song et al. [23] demonstrated that pyrolysis conditions play a decisive role in determining biochar yield and structural characteristics. Fast pyrolysis (50–100 °C/s) achieves higher char yield at elevated temperatures, whereas slow pyrolysis (10 °C/min) produces biochar with a higher specific surface area and enhanced aromaticity. Břendová et al. [24] reported that woody biomass-derived biochar exhibits superior surface area, pore volume, and carbon stability, whereas herbaceous biochar contains more oxygen-containing functional groups. Zhang et al. [25] prepared cotton-stalk biochar via CO_2_ activation combined with nitrogen functionalization, demonstrating that nitrogen incorporation increases surface basicity and adsorption capacity. The adsorption mechanism was found to be temperature-dependent, with physisorption dominating at low temperatures and chemisorption prevailing at elevated temperatures. Zhang et al. [26] reported that ball milling significantly enhances VOC adsorption capacity by increasing surface polarity, introducing functional groups, and improving physicochemical properties. Tseng et al. [27] demonstrated that CuO-loaded activated carbon improves SO_2_ removal through catalytic oxidation to stable sulfate species, enhancing both adsorption efficiency and stability.

**Figure 1:**
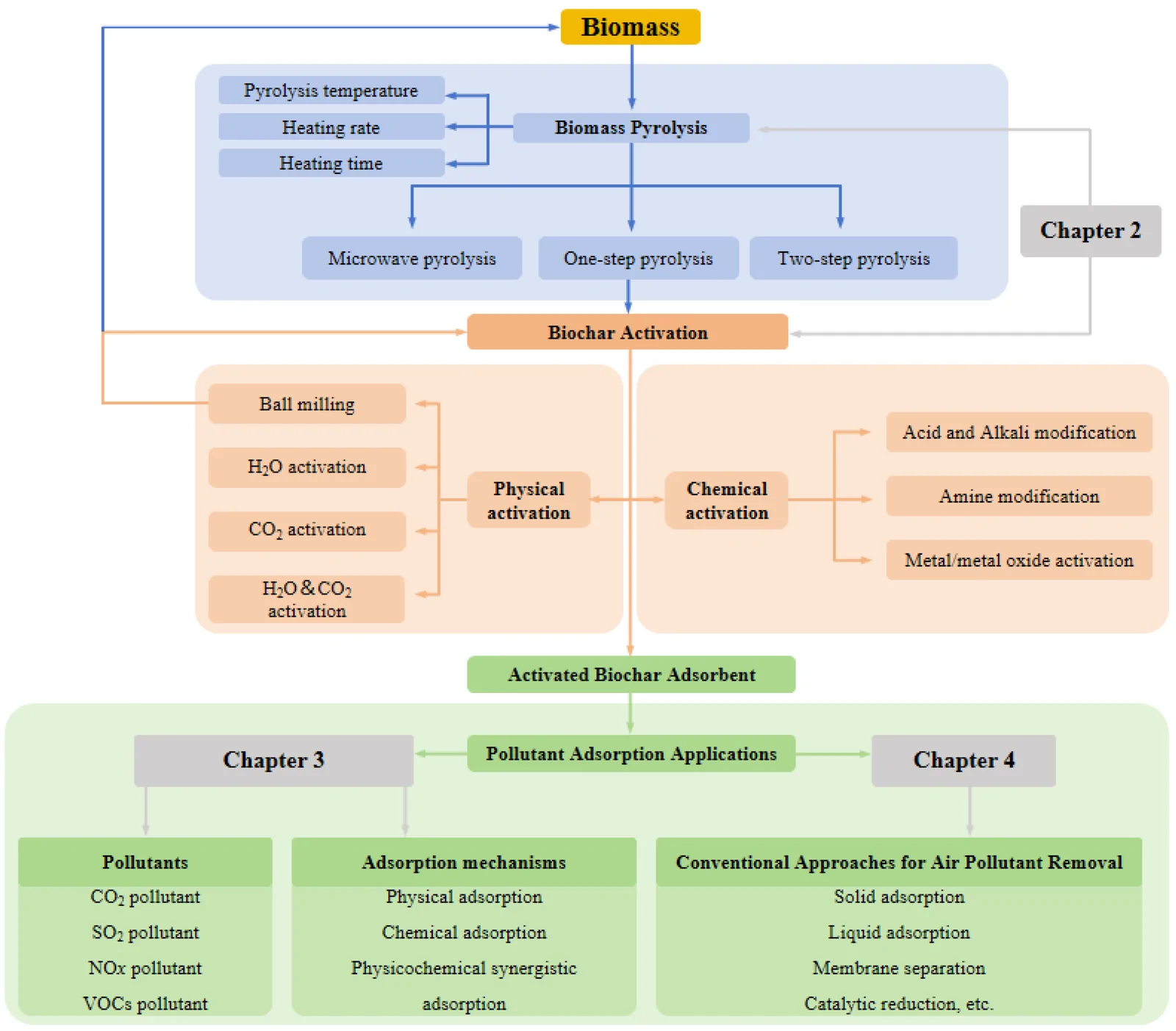
Process flow for the preparation of engineered biochar and a comparison with conventional industrial treatment of polluted gases by adsorption.

As shown in Figure 1, this review focuses on the complete development pathway of engineered biochar. Chapter 2 examines in detail the process of converting biomass feedstock into biochar through pyrolysis and activation, whereby the preparation conditions determine the formation of the pore structure and surface properties. Subsequently, by analyzing the relationship between structural features, surface functional groups and adsorption behavior, the synergistic mechanisms of physical and chemical adsorption are elucidated, and the fundamental adsorption mechanisms governing the capture of gaseous pollutants are explored, including the roles of microporous structures, heteroatom-containing functional groups and metal active sites. Finally, the application of engineered biochar for specific pollutants and its performance compared to conventional adsorption methods are discussed, and the removal efficiency, feasibility and engineering application potential of engineered biochar in removing CO_2_, SO_2_, NO*x* and VOCs are evaluated.

In recent years, research on the removal of gaseous pollutants via adsorption using biochar has been reviewed on numerous occasions; however, the existing literature remains fragmented in several respects. Firstly, most reviews limit their scope to a single gas or a group of gases with similar chemical properties, such as CO_2_ [28], VOCs [29,30], H_2_S [31] and NH_3_ [32]. Secondly, while the majority of reviews describe the methods used to modify and activate biochar, their summaries are often confusing and fail to outline the specific properties of the biochar following modification and activation [33,34]. Finally, while most existing reviews summarize the regeneration capacity of biochar, they do not compare it with conventional flue gas treatment methods or common solid adsorbents. This paper provides a systematic overview of common methods for the preparation of biochar and advanced modification strategies, including physical and chemical activation, microwave treatment, ball milling, and the introduction of functional groups (such as metal loading and amine grafting). It demonstrates how these methods can endow biochar with specific characteristics that enable it to adsorb certain gases more effectively. Subsequently, the paper analyzes the adsorption behavior of common pollutant gases and outlines how conventional biochar can be modified to adsorb these gases. Finally, biochar is compared with conventional pollution treatment technologies and common solid adsorbents. These results demonstrate the potential of engineered biochar as a low-cost, sustainable alternative for controlling industrial gaseous pollutants.

## 2. Pyrolysis and Activation of Biochar

The complex, heterogeneous structure of biochar makes it difficult for traditional characterization methods to fully elucidate its microscopic mechanisms and functional properties. Elemental analysis, specific surface area measurement (BET) and conventional Fourier transform infrared spectroscopy (FTIR) have become standard tools for biochar characterization. These macroscopic or averaged methods often obscure the chemical heterogeneity and pore connectivity of biochar at the nanoscale. Currently, although a range of modern characterization methods based on synchrotron radiation, high-resolution mass spectrometry and advanced microscopy techniques have been developed, they have not yet become widely adopted in the field of biochar research. These cutting-edge techniques enable in-depth analysis of the surface chemical restructuring, pore network topology and interfacial reaction kinetics of biochar, providing key evidence from the molecular to the nanoscale for understanding its environmental behavior. Synchrotron soft/hard X-ray spectroscopy and microscopy techniques are powerful tools for revealing the micro-regional chemical heterogeneity of biochar. Scanning transmission X-ray microscopy–near-edge absorption fine structure (STXM-NEXAFS) is capable of obtaining functional group fingerprints and valence state information for elements such as carbon, nitrogen, oxygen and phosphorus at sub-micrometer resolution. Unlike traditional bulk characterization methods, STXM-NEXAFS can distinguish the localized distribution of graphitised regions, oxygen-containing functional groups and mineral–organic composites. It is particularly well-suited to analyzing the surface chemical restructuring of biochar during soil aging or leaching processes, as well as the bonding environments of nutrient elements. With regard to three-dimensional topography and pore structure characterization, synchrotron nano-CT and phase-contrast CT technologies provide the capability to non-destructively reconstruct pore networks at the nanometer to micrometer scale. Nano-CT can quantify the ratio of closed to open pores and establish the relationship between pore structure and mass transfer/adsorption kinetics, which is not achievable using traditional gas adsorption methods. Focused ion beam–scanning electron microscopy (FIB-SEM) 3D reconstruction techniques, on the other hand, provide a visual representation of biochar’s layered structure, inclusions and mineral-filled pores at nanometer resolution, making them suitable for comparing structural evolution before and after adsorption. In combination with small-angle X-ray scattering (SAXS/SANS) techniques, researchers can statistically analyze the distribution of nanoscale pores, fractal characteristics and pore wall roughness. SANS offers unique advantages for comparison experiments involving hydrogen-containing phases (such as pore water) and must be used in conjunction with adsorption isotherms to infer the true pore structure.

### 2.1. Pyrolysis of Biochar

**Figure 2:**
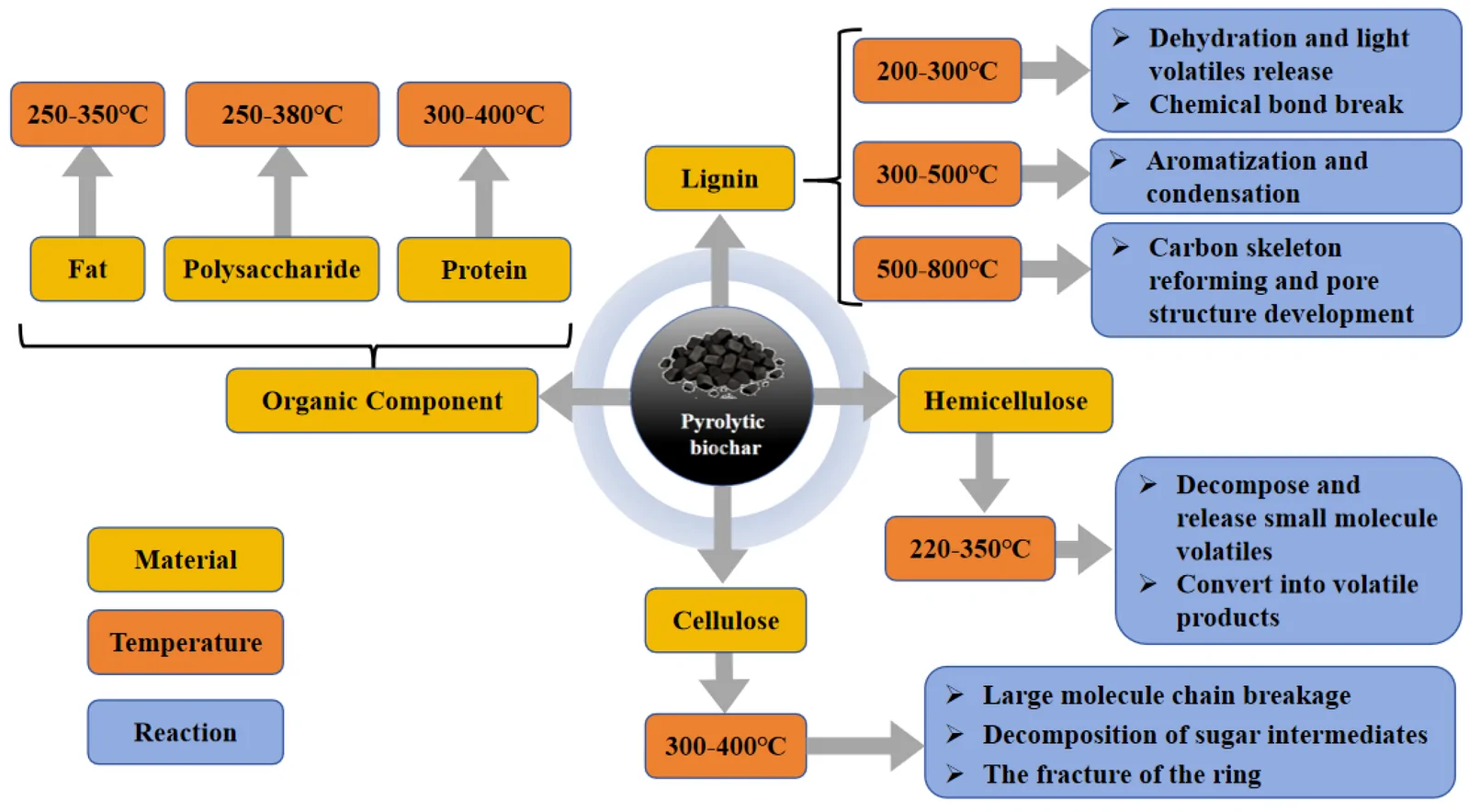
Primary reactions involved in biochar pyrolysis.

Pyrolysis is a thermochemical decomposition process of biomass under oxygen-limited conditions, typically occurring at temperatures of 200–900 °C, producing biochar, bio-oil, syngas, and energy. The physicochemical properties of biochar are primarily governed by feedstock composition, modification strategies, and pyrolysis conditions. The raw material plays a decisive role in the pore structure and surface functional groups of biochar. Cellulose-rich biomass materials tend to produce biochar with a more developed micropore structure, while a higher lignin content at higher pyrolysis temperatures helps to increase micropore volume and aromaticity. During the pyrolysis of cellulose, long polymer chains are broken down into smaller fragments through thermal cracking, after which the resulting sugar intermediates are further decomposed via ring-opening and rearrangement reactions. The oxygen-containing functional groups are released in the form of water and carbon dioxide, forming a carbon skeleton [35]. The decomposition of hemicellulose occurs primarily at relatively low temperatures (220–350 °C), most of it is converted into volatile products, including short-chain hydrocarbons and oxygen-containing organic compounds [36]. Lignin undergoes initial degradation at lower temperatures through the cleavage of ether bonds (e.g., C–O–C linkages), releasing methoxy-substituted aromatic compounds such as guaiacol, isoeugenol, and vanillin. With increasing temperature, side-chain groups (e.g., methoxy groups) are progressively removed via dealkylation reactions, and a corresponding increase in thermally stable aromatic derivatives, such as 2,3-dihydrobenzofuran, occurs. Under moderate to high temperatures, extensive aromatisation and condensation reactions occur predominantly, resulting in the formation of cyclic polyaromatic structures. Ultimately, the residual lignin undergoes further condensation and structural rearrangement, forming stable graphitic-like carbon structures [37]. Figure 2 primarily illustrates the main reactions involved in the pyrolysis of biochar and the products formed at different temperatures.

For biomass with high ash content, pyrolysis typically involves two distinct stages. The first stage (200–400 °C) corresponds to the decomposition of organic components: lipids decompose at 250–350 °C, polysaccharides at 250–380 °C, and proteins at 300–400 °C. These components account for the majority of the mass loss in this stage. The second stage (400–800 °C) involves aromatization and structural stabilization: aliphatic side chains are cleaved, and aromaticity progressively increases. At temperatures above 600 °C, the aromatic rings begin to condense, resulting in a reduction in the rate of mass loss and an increase in the structural stability of the carbon matrix. The inorganic minerals remain largely undecomposed and become components of the biochar [38]. An excessively high ash content in the biomass can have an adverse effect on the textural properties of the biochar. Research by Břendová et al. [24] has demonstrated that an increase in the mineral content of ash reduces the specific surface area of biochar; the melted ash within the biochar fills its pores, reducing its overall pore volume and specific surface area. The textural properties of biochar derived from pyrolysis exhibit substantial variability. Typically, the specific surface area, total pore volume, micropore volume, and mesopore volume range from 8 to 132 m^2^/g, 0.016–0.083 cm^3^/g, 0.012–0.060 cm^3^/g (median: 0.024 cm^3^/g), and 0.007–0.020 cm^3^/g (median: 0.009 cm^3^/g), respectively. Micropores contribute approximately 12.1–58.0% of the total pore volume, while mesopores account for 18.9–31.7% [39].

During pyrolysis, the pyrolysis temperature and heating rate have a significant impact on the pore structure of biochar, determining its adsorption capacity for gaseous pollutants. At relatively low temperatures, biochar primarily develops micropores, whereas higher temperatures promote the formation of mesopores and macropores [40]. An increase in pyrolysis temperature leads to more intense decomposition of the organic components, increasing the specific surface area and porosity [41]. However, excessively high temperatures may cause pore collapse and structural degradation, reducing the adsorption capacity for certain pollutants [39]. The heating rate also determines the properties of biochar. Rapid pyrolysis helps to form a microporous structure and increase the specific surface area, but it can also lead to structural instability in the biochar [23]. Slow pyrolysis produces a more stable carbon structure and a more uniform pore distribution, but the specific surface area of the resulting biochar is relatively lower than that of biochar produced by rapid pyrolysis [42].

Conventional one-step pyrolysis is the most widely used method. It works by transferring heat from an external heat source to the biomass under oxygen-deficient conditions, triggering thermal decomposition [43]. As the temperature rises during this process, the primary organic components of the biomass (including hemicellulose and cellulose) undergo thermal decomposition, producing volatile gases, tars (precursors of bio-oil), and solid carbon residues. These subsequently undergo secondary reactions in the high-temperature environment, involving dehydration, decarboxylation, and decarbonylation reactions, ultimately forming a stable, carbon-rich structure.

Microwave pyrolysis is a direct heating method using microwave radiation. Unlike conventional external heating, polar molecules within the biomass (such as water and oxygen-containing functional groups) rapidly rotate and vibrate under the influence of the electromagnetic field, converting microwave energy into thermal energy and achieving rapid internal heating [44]. This internal heating mechanism enables faster heating rates, more uniform temperature distribution, and higher energy efficiency. The dielectric properties of biomass determine its microwave absorption capacity. Generally, the adsorption capacity is related to temperature and composition [45]. Many biomass materials have limited microwave absorption capacity; therefore, microwave-assisted heating agents (such as biochar, carbon materials, and metal oxides) are often employed to absorb microwave energy and transfer heat to the biomass via conduction and convection, enhancing heating efficiency and the achievable temperature [46]. The decomposition pathways in microwave pyrolysis are similar to those in conventional pyrolysis, both involving the decomposition of hemicellulose, cellulose, and lignin to produce biochar, bio-oil, and syngas. Notably, microwave pyrolysis can reduce the residence time of volatile intermediates, suppressing secondary reactions and potentially increasing the yield of gaseous products [47].

**Figure 3:**
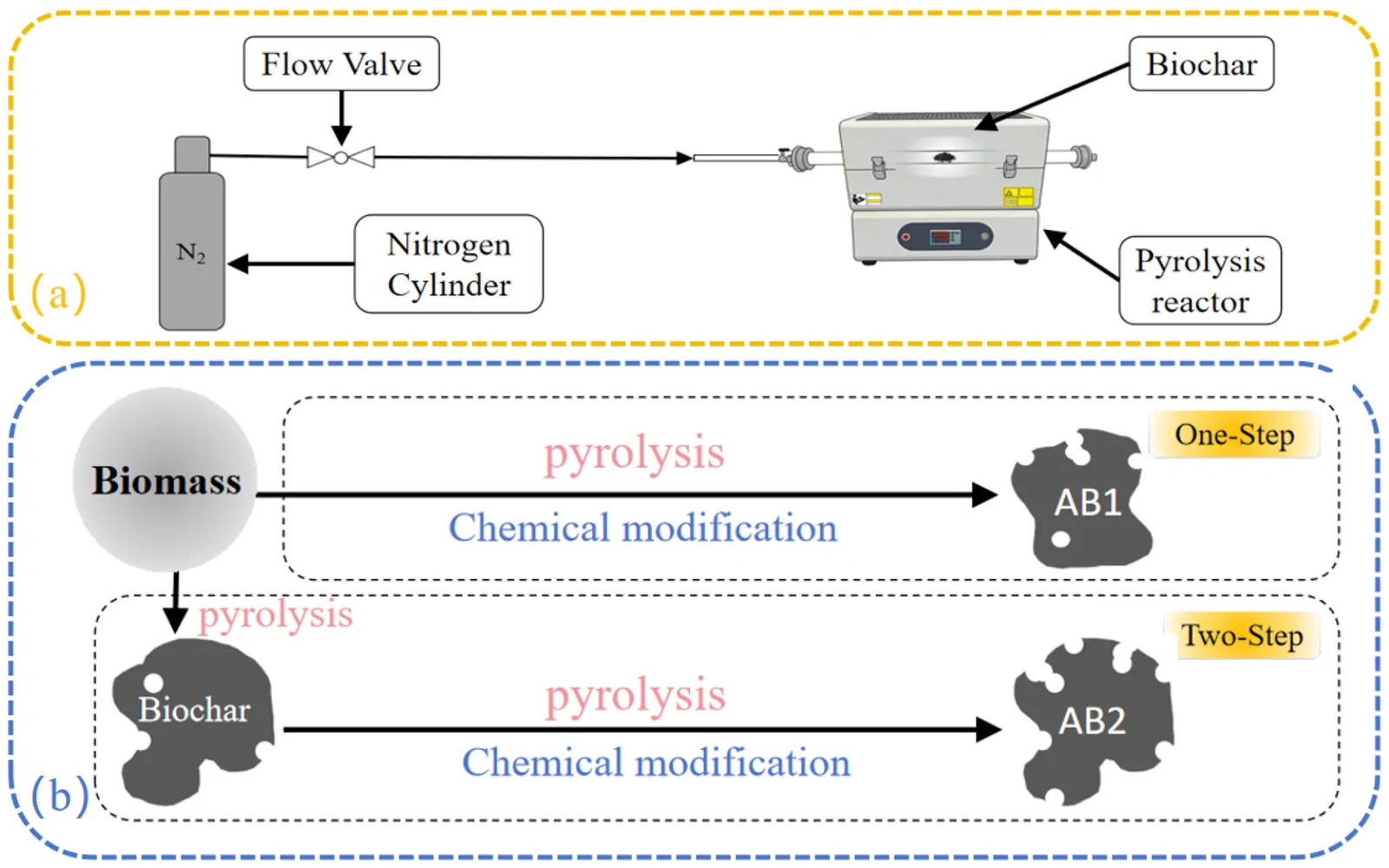
(**a**) Pyrolysis flow chart; (**b**) flow charts of one-step and two-step pyrolysis methods.

Two-step pyrolysis is a pyrolysis method involving staged thermal treatment, typically comprising a pre-treatment (low-temperature pyrolysis) stage followed by high-temperature pyrolysis or activation. This method allows for more precise control of the pore structure of biochar, enhancing its ability to adsorb specific gaseous pollutants. For example, Fu et al. [48] compared one-step and two-step pyrolysis processes for the preparation of rice husk biochar. The two-step method (KOH: biochar = 1:1) achieved a significantly higher yield (19.4%) than the one-step method (2.5%), an increase of 7.76 times. This enhancement can be attributed to the decoupling of carbonization and activation in the two-step method. This separation can prevent excessive carbon consumption by KOH, leading to an increase in char yield. Furthermore, the formation of the stable carbon framework during the two-step pyrolysis is beneficial to the development of the adsorption capacity. The optimal sample obtained via two-step pyrolysis (AB2-3-750) exhibited a phenol adsorption capacity of 201 mg/g, which was 2.43 times higher than that of the sample prepared by one-step pyrolysis (82.5 mg/g). As this method involves more steps than the conventional single-step pyrolysis process, it is more labor-intensive. Whilst there are existing publications that have utilized this method, its application remains limited. Furthermore, there are no published studies comparing how much the specific surface area and adsorption capacity can be increased through this method, leaving scope for further research. Figure 3 summarises and illustrates the experimental flowcharts for these two methods.

### 2.2. Biochar Activation

Biochar activation methods are generally classified into two categories: physical activation and chemical activation. Physical activation typically involves the use of oxidizing agents, such as carbon dioxide, steam, or air at high temperatures (350–1000 °C), to remove residual volatile compounds and tar, etch the carbon framework of the biochar, increase the specific surface area and promote the formation of a pore structure [12,49]. Chemical activation, on the other hand, employs chemical reagents (such as KOH, NaOH, ZnCl_2_, KHCO_3_, and K_2_CO_3_). These reagents react with the carbon matrix to form additional pore structures through a chemical etching process, typically carried out at relatively lower temperatures (600–900 °C) [25,50,51,52].

Physical modification alone optimizes the pore structure but lacks chemically active sites, resulting in poor selectivity for the adsorption of pollutants in high-temperature flue gas. Chemical modification alone tends to clog the micropores, compromising physical adsorption capacity and leading to only a limited increase in total adsorption capacity. Both physical and chemical modifications are typically employed in the preparation of functional biochar.

#### 2.2.1. Physical Treatment

Ball milling is an effective mechanical modification strategy to optimize the properties of biochar [53]. It is generally classified as a physical modification method, since it barely alters the bulk chemical composition of biochar. During high-energy collisions in the milling process, kinetic energy is transferred to biochar particles, inducing partial bond cleavage, particle refinement to the nanoscale (<1000 nm), lattice distortion, and increased structural defects. These changes collectively create an abundance of active surface sites [54]. First, ball milling significantly enhances specific surface area and improves pore accessibility, providing more active adsorption sites [55]. Raczkiewicz et al. [56] reported that following ball milling, nanoscale biochar derived from willow exhibited substantial increases in surface area, from 7.2 to 146.3 m^2^/g at 450 °C, from 3.9 to 160.8 m^2^/g at 750 °C, and from 3.7 to 158.6 m^2^/g for rice husk-derived biochar at 550 °C. These results indicate that nanosized biochar exhibits a higher surface expansion coefficient and enhanced interaction with pollutants. Second, ball milling increases the abundance of oxygen-containing functional groups (e.g., –OH and –COOH), strengthening interactions with pollutants through hydrogen bonding and electrostatic interactions. Wang et al. [57] reported that ball milling increased the content of surface functional groups (–OH, C=C, and C–O) and aromatic structures, improving the removal efficiency of methylene blue (MB). Furthermore, nanoscale particle size enhances dispersion and contact efficiency between biochar and pollutants. In addition, the exposure of aromatic domains promotes π–π interactions with organic pollutants [58]. Ball-milled biochar has also demonstrated strong performance in heavy metal adsorption [59]. The ball milling method is environmentally friendly and economical because the process usually requires only a small amount or no solvent at all, so as to minimize chemical pollution, and its equipment structure is relatively simple, easy to operate, and maintain [56]. Furthermore, the ball-milling method enhances the adsorption and catalytic properties of biochar by significantly increasing its specific surface area, introducing oxygen-containing functional groups, and reducing particle size to the nanoscale.

Carbon dioxide activation is an effective method for enhancing the adsorption performance of biochar and improving its pore structure [60]. As a mild oxidizing agent, carbon dioxide is able to penetrate the pore network of biochar and react with the carbon, enlarging existing pores and generating new ones [61,62]. Typically, pre-treated biochar is activated in a carbon dioxide atmosphere at elevated temperatures (600–1200 °C) [63]. The formation of micropores during the carbon dioxide activation process is primarily attributed to the gasification reaction between carbon and carbon dioxide [64,65], a reaction that significantly increases the porosity and specific surface area of the biochar. In carbon dioxide activation, the pore structure and surface properties of biochar can be effectively controlled by adjusting the activation temperature, activation time, and carbon dioxide flow rate [63]. Excessively high activation temperatures or prolonged activation times may also lead to excessive structural damage to the biochar. The CO_2_ activation conditions need to be optimized [66]. However, excessive temperatures promote pore overlap, carbon framework fragility, and structural collapse.

The physical activation method using carbon dioxide offers several advantages, but it also has certain limitations. On the one hand, carbon dioxide is non-toxic and does not introduce impurities, avoiding secondary pollution caused by residual chemical activators [64]. The biochar produced by this method is typically of high purity and can be directly applied in fields requiring clean materials, such as soil remediation and water treatment. Furthermore, compared to chemical activators (such as KOH and H_3_PO_4_), CO_2_ is non-corrosive to activation equipment (such as quartz or corundum furnaces), which significantly extends the service life of the equipment and reduces maintenance costs [67]. Furthermore, the CO generated during the activation process can be recovered and combusted to provide thermal energy, while unreacted CO_2_ can be purified and recycled as an activator, enhancing the sustainability of the process.

The high energy consumption associated with the carbon dioxide activation process limits its large-scale application [68]. Furthermore, compared with chemical activation, the reaction rate between carbon dioxide and carbon is relatively slow, resulting in prolonged preparation times and reduced carbon yield [69]. Biochar activated by carbon dioxide primarily exhibits a microporous structure, with relatively low proportions of mesopores and macropores. Consequently, carbon dioxide-activated biochar is more suitable for adsorbing small-molecule substances such as VOCs and heavy metal ions [12,70].

Steam activation is another widely used physical activation method, which can effectively increase the porosity and specific surface area. This method is typically carried out in superheated steam at temperatures of 700–900 °C, with activation times as short as 30 min. Due to the small molecular size of H_2_O, steam can rapidly penetrate the porous carbon matrix, significantly promoting pore development [71]. During the steam activation process, carbon reacts with H_2_O to form CO and H_2_ (as shown in Equation (1)), indicating that carbon is consumed while simultaneously generating more micropores and increasing the specific surface area [61]. Furthermore, superheated steam effectively removes volatiles and tar residues left within the pore structure during pyrolysis, further enhancing the porosity of biochar.

C + H_2_O → CO + H_2_ (1)

The presence of CO can accelerate the gasification of carbon by removing the oxygen-containing complexes in the form of CO_2_ on the surface of char. During the activation process, the overall reaction between steam and carbon involves several coupled processes, including the chemical adsorption of water, carbon gasification, and the water-steam conversion reaction. Initially, water molecules (H_2_O) undergo endothermic chemisorption on the carbon surface, forming surface oxygen complexes (C(O)) (Equation (2)), which may further decompose to produce carbon monoxide (CO) (Equation (3)). The presence of CO can accelerate the gasification of carbon by removing the oxygen-containing complexes on the surface of char in the form of CO_2_ (Equation (4)). Subsequently, the exothermic water–gas shift reaction occurs (Equation (5)). Meanwhile, direct gasification of carbon by steam takes place; CO_2_ and H_2_ are generated in this process (Equation (6)). The generated CO_2_ and H_2_ further participate in secondary reactions that activate the carbon surface. Specifically, CO_2_ reacts with carbon to produce CO (Equation (7)), while hydrogen can react with carbon to form methane (CH_4_) (Equation (8)). In addition, CH_4_ can further react with steam to generate CO and H_2_ (Equation (9)). These sequential and parallel reactions collectively lead to the continuous consumption of carbon, promoting the formation and development of pore structures. As a result, steam activation typically produces a broader pore size distribution, enabling the formation of both micropores and mesopores.

C + H_2_(O) → C(O) + H_2_ (2)

C(O) → CO + C(3)

CO + C(O) → CO_2_ + C (4)

CO + H_2_O → CO_2_ + H_2_ (5)

C + 2 H_2_O → CO_2_ + 2 H_2_ (6)

C + CO_2_ → 2 CO (7)

C + 2 H_2_ → CH_4_ (8)

CH_4_ + H_2_O → CO + 3 H_2_ (9)

By adjusting the steam activation temperature, it is also possible to effectively control the pore structure and surface properties of biochar. At temperatures below 750 °C, steam activation primarily promotes the development of micropores, whereas temperatures above 750 °C are beneficial to the formation of both micropores and mesopores [61]. However, excessively high temperatures (e.g., >800 °C) may induce over-gasification, leading to the depletion of small aromatic structures and the formation of more ordered but less reactive carbon frameworks. For example, Yu et al. [71] prepared activated carbon from pinecones via steam activation, obtaining materials with well-developed porous structures.

**Figure 4:**
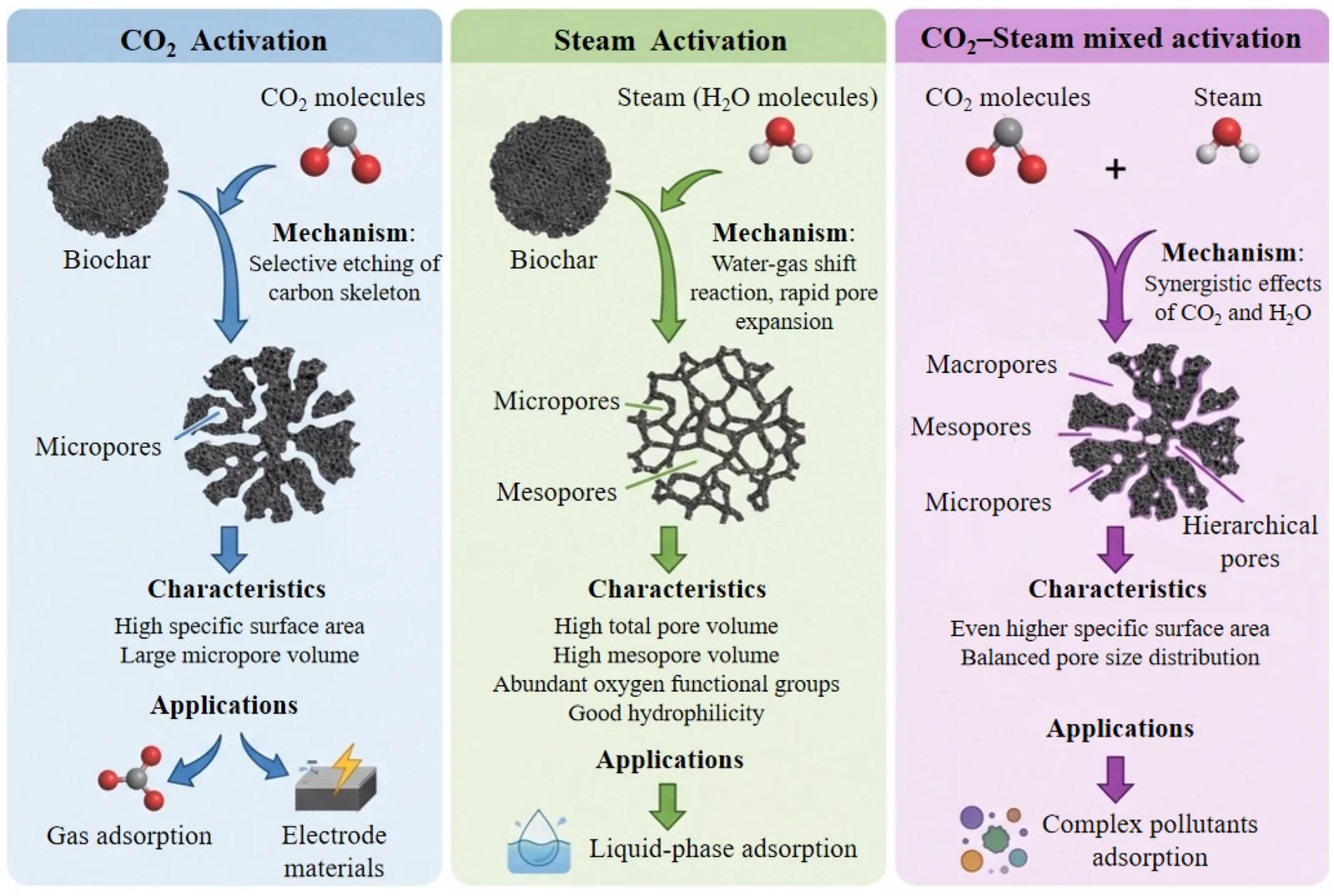
The mechanisms and advantages of CO_2_ activation, steam activation, and their combined activation strategies.

In addition to activation using a single activator, CO_2_–steam mixed activation is also an effective activation method. Liu et al. [72] used Malim wood as feedstock and compared the activation of biochar under pure CO_2_, 15% vol% H_2_O (balanced with Ar), and H_2_O/CO_2_ mixed atmospheres. In H_2_O/CO_2_ mixed activation, the high reactivity of H_2_O plays a dominant role: the high reactivity of H_2_O and its high content of oxygen-containing functional groups result in low selectivity for carbon removal, and the evolution of the pore structure is similar to that observed in H_2_O activation. However, the presence of carbon dioxide further promoted the development of the pore network, resulting in slightly improved structural properties compared to steam activation alone. This hybrid activation system achieves a balance between micropore retention and mesopore formation, while overcoming the limitations of slow reaction kinetics in CO_2_ activation and excessive micropore depletion in steam activation. Consequently, CO_2_–steam co-activation demonstrates superior overall performance in tailoring biochar pore structures and enhancing adsorption properties. Figure 4 outlines the mechanisms of the three methods and their different application scenarios.

#### 2.2.2. Chemical Activation

**Figure 5:**
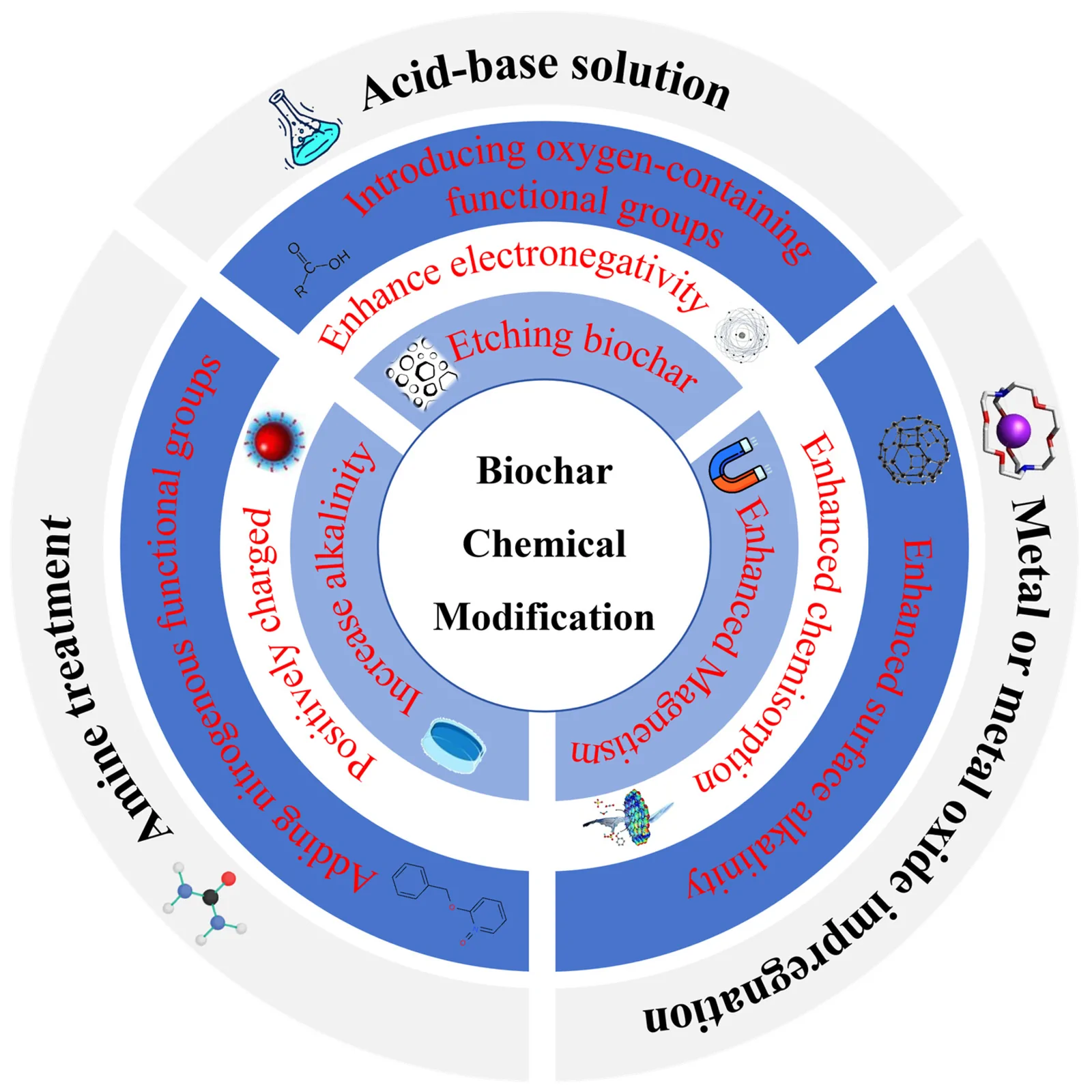
The typical chemical activation methods and their underlying mechanisms.

Chemical modification is widely employed to improve the surface chemical properties of pristine biochar, including surface acidity/basicity, functional groups, hydrophobicity, and polarity. Chemical activation can generally be conducted via two approaches: pre-treatment and post-treatment. In the pre-treatment method, biomass is directly impregnated with chemical reagents prior to pyrolysis, whereas in the post-treatment method, the biochar is subsequently impregnated and thermally treated. Among various chemical modification strategies, acid–base activation [51,73], amine functionalization [74,75,76], and metal or metal oxide impregnation [77,78,79] are widely recognized as the most effective approaches for tailoring the surface chemistry of biochar. Figure 5 illustrates the main mechanisms by which the three methods modify biochar.

Acid modification is an effective method for removing inorganic ash components from biochar, which opens up the pore structure while increasing the specific surface area and the number of available adsorption sites. Furthermore, acid treatment introduces a large number of oxygen-containing functional groups (such as carboxyl groups (–COOH) and hydroxyl groups (–OH)) on the surface of biochar [80]. These functional groups enhance adsorption through electrostatic interactions with positively charged pollutants, while also forming stable complexes via chemical bonding [81]. Consequently, electrostatic attraction is considered one of the primary mechanisms for the adsorption of gaseous pollutants onto acid-modified biochar [82]. Typically, the surface of acid-modified biochar carries a negative charge, particularly when the pH value of the solution exceeds the zero-charge point (pH_p_zc). Under these conditions, stronger electrostatic attraction towards cations can be achieved. It is worth noting that a lower pH_p_zc value generally implies a stronger adsorption capacity for positively charged ions under neutral conditions.

Alkali modification enhances the gas adsorption performance of biochar through two main mechanisms. Firstly, alkaline reagents react with the inherent surface functional groups of biochar (such as –COOH, –OH, and C=O) to generate stronger basic sites, such as phenolate anions (–O^−^), amine groups (–NH_2_, –NH–), nitrogen-containing heterocycles (such as pyridine and pyrrole), and metal oxide or hydroxide sites. Secondly, the corrosive nature of alkaline reagents (e.g., KOH and NaOH) effectively etches amorphous carbon and mineral components (e.g., SiO_2_ and Al_2_O_3_), removing impurities and opening up blocked micropores and mesopores [83]. At high temperatures (600–800 °C), alkaline reagents react with the carbon matrix, leading to pore expansion, the conversion of micropores into mesopores, and the formation of a new pore structure. Consequently, the specific surface area of biochar can be significantly increased, typically rising from 100 to 300 m^2^/g to 1000–2000 m^2^/g [83]. The optimized pore structure provides ample space for the physical adsorption of acidic gas molecules, while reducing diffusion resistance. Acidic gases (such as SO_2_ and CO_2_) initially diffuse through macropores (>50 nm), subsequently migrate to mesopores, and are adsorbed within the micropores. Upon reaching the biochar surface, acidic gas molecules are first adsorbed via physical interactions such as van der Waals forces and dipole–dipole interactions. Polar acidic gases (such as SO_2_ and NO_2_) have relatively large molecular dipole moments (SO_2_: 1.63 D; NO_2_: 0.31 D), strong dipole–dipole interactions with basic functional groups (e.g., –O^−^ and –NH_2_) facilitate initial adsorption. The diversity of surface functional groups not only increases the density of active sites but also optimizes diffusion pathways within the mesopore and micropore structures, simultaneously enhancing both adsorption capacity and adsorption kinetics [84].

2 NaOH + SiO_2_ = NaSiO_3_ + H_2_O (10)

Studies have shown that nitrogen-containing functional groups can significantly enhance the adsorption performance of biochar, particularly its adsorption capacity for gaseous pollutants [85]. Nitrogen-containing functional groups such as pyridine, amide, imide, lactam, and pyrrole can effectively increase the surface alkalinity of biochar [86]. These nitrogen-containing functional groups are typically introduced via nitrogen-rich precursors, including ammonia (NH_3_), amines, nitric acid (HNO_3_), melamine, and polyacrylonitrile [87,88]. Surface amination is currently one of the most widely used strategies, involving the introduction of amino groups (–NH_2_) onto the surface of biochar. Three main methods are commonly employed: (i) ammonia treatment, (ii) direct grafting using amine-containing reagents, and (iii) a combined nitration-reduction process [89,90].

In nitrification processes using NH_3_, as the reaction between ammonia and carboxyl groups is highly reactive, biochar is typically pre-oxidized to activate its surface, promoting the reaction with NH_3_ [73,91]. For instance, nitric acid pre-oxidation introduces abundant carboxyl groups onto the biochar surface. Subsequent reaction with NH_3_ triggers a dehydration (decarboxylation) reaction, forming amide, imide (formed from two carboxyl groups), and lactam functional groups. Further heat treatment (e.g., treatment at approximately 200 °C for 2.5 h) promotes dehydration, decarboxylation, and decarbonylation reactions, facilitating the formation of heterocyclic nitrogen structures such as pyrrole and pyridine [92]. Furthermore, heat treatment induces condensation reactions between surface functional groups, further optimizing the surface chemical properties.

Amination introduces amino groups (–NH_2_), which enhance adsorption capacity through acid–base interactions or by forming chemical bonds with gaseous pollutants such as carbon dioxide (CO_2_), nitric oxide (NO*x*), and sulfur dioxide (SO_2_). Due to their high reactivity and ease of availability, low-molecular-weight amines (such as diethylamine (DEA) and tetraethylpentaamine (TEPA)) are widely used [93]. To achieve effective amine grafting, the biochar surface must contain oxygen-containing functional groups, such as hydroxyl (–OH), carbonyl (C=O), and, in particular, carboxyl (–COOH) groups. However, unactivated carboxyl groups exhibit insufficient reactivity towards amines. A widely used activation strategy involves 1-ethyl-3-(3-dimethylaminopropyl) carbodiimide (EDC) and hydroxybenzotriazole (HOBt). Mechanistically, EDC first reacts with the carboxyl group via proton abstraction to form an O-acyl isourea intermediate. Subsequently, HOBt undergoes a nucleophilic substitution reaction on the activated electrophilic carbon, displacing the intermediate and forming a more stable active ester, while suppressing side reactions (such as the formation of urea). This activation step significantly enhances the efficiency of amination. Following activation, amine compounds such as tetraethylenepentaamine (TEPA) undergo a nucleophilic attack on the electrophilic carbon sites on the biochar via the lone pair of electrons on their nitrogen atoms, forming amine-functionalised biochar (amide structure) and regenerating HOBt. TEPA amination process uses HOBt as stabilizer. The introduction of amino groups significantly increases the density of basic active sites on the biochar surface, enhancing its adsorption capacity for acidic gases such as carbon dioxide. In addition to direct amination, nitrogen-containing functional groups can also be introduced via a nitration-reduction pathway, which is primarily based on electrophilic aromatic substitution reactions. This method enables the incorporation of nitrogen-containing groups into the polycyclic aromatic carbon framework of biochar [94].

The combination of nitration and reduction is an effective strategy for the targeted introduction of nitrogen-containing functional groups into the polycyclic aromatic structures of biochar. Its fundamental principle is based on the mechanism of electrophilic aromatic substitution. This method achieves the stepwise introduction of amino groups (–NH_2_) by first subjecting the aromatic precursor to nitration, followed by the reduction. In the nitration step, concentrated nitric acid or a mixed acid (HNO_3_/H_2_SO_4_) is typically used to react with the condensed aromatic skeleton of biochar at 50–100 °C. The nitrite ion (NO_2_^+^), acting as a strong electrophile, attacks electron-rich sites on the aromatic ring, replacing hydrogen atoms and forming biochar containing nitro functional groups, which serves as a precursor for subsequent amination [95,96]. X-ray photoelectron spectroscopy (XPS) analysis indicates that this strategy can increase the nitrogen content of biochar from approximately 0.71% to over 1.5%, with heterocyclic nitrogen species (such as pyridine nitrogen and pyrrole nitrogen) accounting for more than 60% of the total nitrogen content [89]. These nitrogen functional groups are linked to the aromatic skeleton via covalent bonds and exhibit high thermal stability. Their thermal stability is far superior to that of physically adsorbed amine compounds. Even after regeneration at temperatures exceeding 400 °C, their retention rate remains above 80%. The introduction of amino and pyridine nitrogen groups not only increases the density of basic sites on the biochar surface but also enhances its electron-donating capacity. Consequently, through acid–base interactions and chemical bonding, the adsorption capacity for acidic gases such as CO_2_ and SO_2_ has been significantly enhanced [97].

The incorporation of nitrogen-containing functional groups markedly improves the adsorption performance of biochar toward gaseous pollutants [12,98]. Molecular simulation studies have shown that pyridinic and pyrrolic nitrogen species enhance the adsorption of typical VOCs, such as toluene, phenol, and chlorobenzene, primarily by modifying surface electrostatic potential and strengthening non-covalent interactions [85]. Furthermore, nitrogen functionalities also play a crucial role in heavy metal adsorption. Density functional theory (DFT) calculations indicate that nitrogen-doped biochar exhibits enhanced binding affinity toward lead species (Pb^0^, PbO, and PbCl_2_) [99]. To further improve nitrogen incorporation efficiency, Chatterjee et al. [93] developed a dual amination strategy assisted by low-frequency ultrasonic irradiation. The ultrasonic treatment exfoliates graphitic domains, generates additional micropores, and opens blocked pore channels, significantly enhancing functionalization efficiency. This approach, which combines physical modification with chemical amination, enables the preparation of nitrogen-rich adsorbents with superior carbon dioxide capture performance, highlighting the effectiveness of physically assisted chemical functionalization. Furthermore, the ammonia treatment increased the porosity and pore volume, further enhancing the adsorption performance.

Nitrogen functionalization may lead to pore blockage, significantly reducing the specific surface area and pore volume. Du et al. [100] reported that biochar derived from soya bean meal exhibited a high specific surface area of 1589.35 m^2^/g following KOH activation. However, subsequent nitrogen doping with melamine resulted in a 42.67% reduction in specific surface area (to 911.10 m^2^/g). Although the nitrogen content increased from 0.47% to 6.15%, the CO_2_ adsorption capacity did not increase proportionally. This is mainly because CO_2_ adsorption is dominated by physical adsorption. The decline in specific surface area due to pore blockage after nitrogen functionalization consequently reduces the CO_2_ adsorption capacity. To address this issue, nitrogen-rich biomass precursors can be utilized directly to achieve in situ self-doping of nitrogen during the pyrolysis process, avoiding pore blockage. For example, Du et al. [19] employed pomelo peel as a nitrogen-rich precursor and activated it via ZnCl_2_ impregnation, achieving CO_2_ adsorption capacities of 3.01 mmol/g (25 °C, 1 bar) and 4.83 mmol/g (0 °C, 1 bar), demonstrating the effectiveness of this strategy.

**Figure 6:**
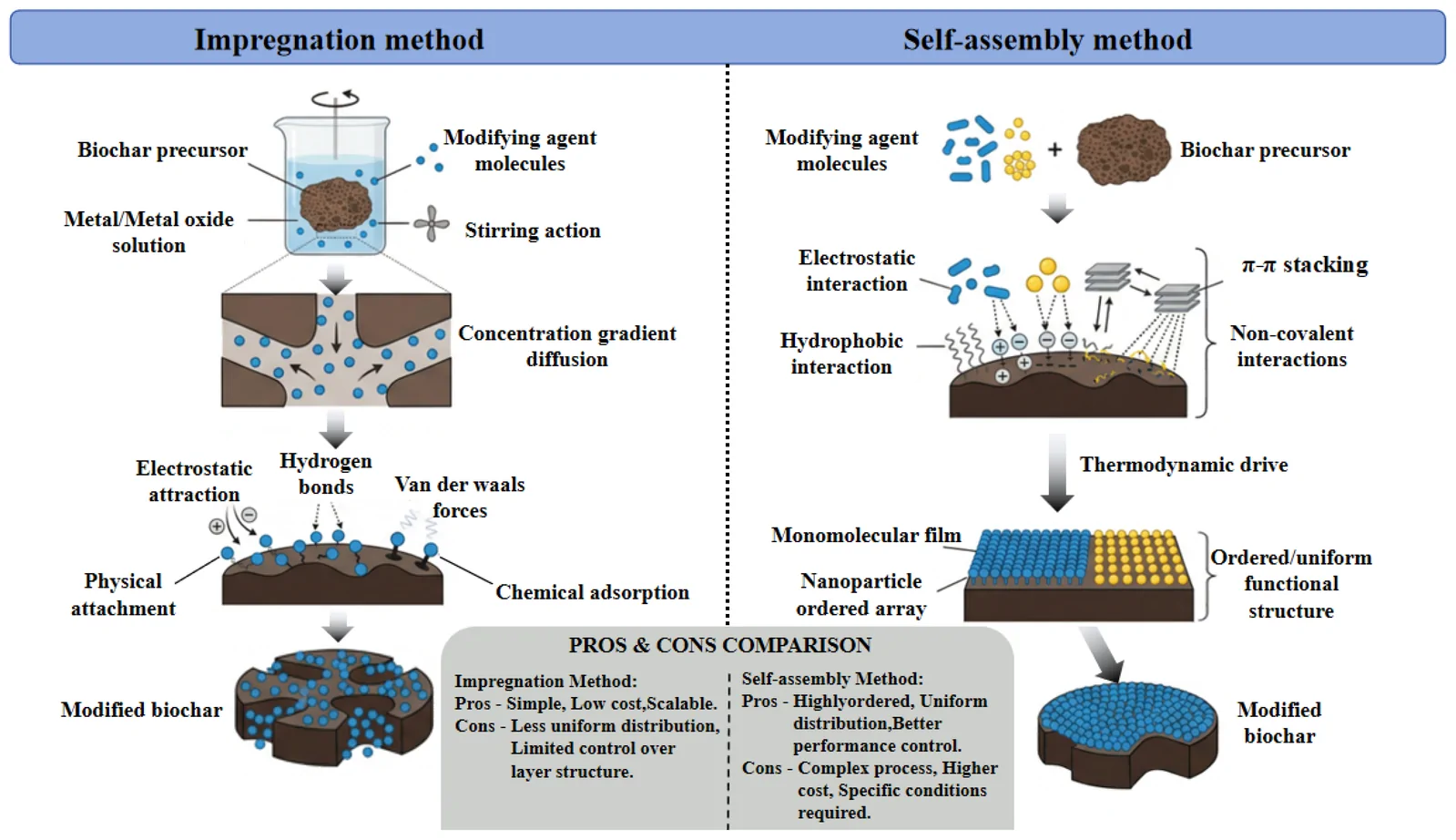
Schematic illustration of impregnation and self-assembly mechanisms.

Modifying biochar using metals or metal oxides is also a common and effective method. This involves first impregnating the biochar with a solution of metal salts (such as AlCl_3_ [101], MgCl_2_ [102], and FeCl_3_ [103]), followed by pyrolysis to convert them into the corresponding metal oxides. Furthermore, metal oxides can also be used directly as precursors for biochar modification [18,104]. Among various preparation methods, impregnation is the most widely employed approach [105]. This method typically involves soaking biochar precursors in metal or metal oxide solutions while stirring. The attachment of metal ions is primarily driven by concentration gradients and interfacial interactions, including electrostatic attraction, hydrogen bonding, and van der Waals forces, leading to physical attachment or chemical anchoring of active species to the biochar surface and within its pore structure. Figure 6 outlines the mechanisms and the advantages and disadvantages of the impregnation and self-assembly methods.

In contrast, the self-assembly method represents a more advanced modification strategy [106]. Rather than relying on external agitation, it spontaneously forms an ordered, uniform functional structure driven by thermodynamics through the synergistic action of non-covalent bonds between modifier molecules or nanoparticles and the biochar surface, as well as between the molecules themselves. The core of this method lies in ‘spontaneous ordering’, whereby specific structures are formed through the oriented arrangement of building blocks based solely on intrinsic interactions between them, without the need for external forces. Self-assembly offers superior control over functional site distribution and structural uniformity, as well as improved stability and resistance to leaching. Experiments on this method have been reported in the literature. However, no comparison has been made between the two methods. Further research into their performance and cost-effectiveness may be conducted in the future. Impregnation remains the dominant method due to its simplicity, cost-effectiveness, and suitability for large-scale industrial applications.

The incorporation of metal oxides increases the surface alkalinity of biochar, enhancing its affinity for acidic gases such as carbon dioxide, sulfur dioxide, and nitrogen oxides [107,108]. For example, Lahijani et al. [109] impregnated biochar prepared at 900 °C with an alkali metal solution, followed by heat treatment in a nitrogen atmosphere at 500 °C. This process allowed metal active sites to be incorporated into the carbon matrix, enhancing the adsorption capacity of metal-loaded biochar for acidic CO_2_. The adsorption capacities of biochar modified with different metals exhibit the following trend: Mg > Al > Fe > Ni > Ca > raw biochar > Na. Among these, Mg-loaded biochar performed most effectively, with a CO_2_ adsorption capacity of 82.0 mg/g, which is significantly higher than that of raw biochar (72.6 mg/g) and other metal-modified samples, representing an increase of approximately 15.7%.

Mg-loaded biochar demonstrated excellent cyclic stability, maintaining its CO_2_ capture capacity over multiple adsorption–desorption cycles, along with favorable regeneration ability and rapid desorption kinetics. The enhanced performance can be attributed to the formation of MgO via thermal decomposition of MgCl_2_·6H_2_O (Equations (11)–(13)). The basic sites (O^2−^) on MgO interact with acidic CO_2_ molecules to form carbonate intermediates, strengthening adsorption interactions. However, despite its high performance, the relatively high cost of Mg limits its large-scale application, especially given that improvements in adsorption capacity are generally within 20%. In contrast, Fe-based modifiers offer a more cost-effective alternative while still providing satisfactory enhancement in adsorption performance. Consequently, iron salts such as FeCl_3_ and Fe(NO_3_)_3_ are more commonly employed for biochar modification in practical applications.

MgCl_2_·6H_2_O → MgCl_2_·2H_2_O + 4 H_2_O (11)

MgCl_2_·2H_2_O → Mg(OH)Cl + H_2_O + HCl (12)

Mg(OH)Cl → MgO + HCl(13)

The metal modification of biochar is influenced not only by the intrinsic properties of the metal cations but also by the type of accompanying anions in the precursor salts. Zubbri et al. [110] employed a wet impregnation method, dispersing 0.5 g of biochar in 60 mL of a 5 wt% solution of magnesium salts (Mg(NO_3_)_2_, MgSO_4_, MgCl_2_ and Mg(CH_3_COO)_2_), followed by drying and heat treatment at 500 °C under a nitrogen atmosphere to form MgO. The results indicate that the modification effect depends largely on the anion type, with the following order of effectiveness: Mg(NO_3_)_2_ > MgSO_4_ > MgCl_2_ > Mg(CH_3_COO)_2_. Among these, Mg(NO_3_)_2_-modified biochar exhibited the highest CO_2_ adsorption capacity (76.78 mg/g). This superior performance is attributed to the complete thermal decomposition of magnesium nitrate into MgO at approximately 500 °C, providing abundant basic active sites. In contrast, magnesium acetate undergoes only incomplete decomposition, leaving residual MgCO_3_, and consequently generates fewer active sites.

The thermal decomposition of magnesium acetate occurs in several stages. First, it undergoes dehydration to form anhydrous magnesium acetate; subsequently, it decomposes into magnesium carbonate (MgCO_3_) and acetone (CH_3_COCH_3_); finally, upon prolonged heating, it is converted into magnesium oxide (MgO). By contrast, at around 400 °C, magnesium nitrate decomposes more efficiently into MgO via a two-step process.

Mg(CH_3_COO)_2_·4H_2_O → Mg(CH_3_COO)_2_ + 4 H_2_O ↑ (14)

Mg(CH_3_COO)_2_ → MgCO_3_ + CH_3_COOH_3_ ↑ (15)

MgCO_3_ → MgO + CO_2_ ↑ (16)

Mg(NO_3_)_2_·6H_2_O → Mg(NO_3_)_2_ + 6H_2_O ↑ (17)

Mg(NO_3_)_2_ → MgO + 2 NO_2_ ↑ + O_2_ ↑ (18)

During the metal modification process, it is essential to precisely control the metal loading and precursor concentration. Insufficient metal content or excessively low solution concentration may result in poor modification efficiency, leading to a low increase in adsorption capacity. Conversely, excessively high metal loading or concentration may cause structural damage to the carbon framework due to excessive etching effects or lead to the blockage of the biochar pore structure, reducing the final adsorption capacity [110]. Modification methods typically affect the regeneration properties of biochar. For example, nitrogen-doped biochar retains approximately 85% of its carbon dioxide adsorption capacity after 10 adsorption–desorption cycles, while biochar composites doped with metal hydroxides (such as Al, Fe, and Mg) maintain a capacity of over 90% following regeneration at 120 °C.

## 3. Adsorption Mechanisms of Atmospheric Pollutants

**Figure 7:**
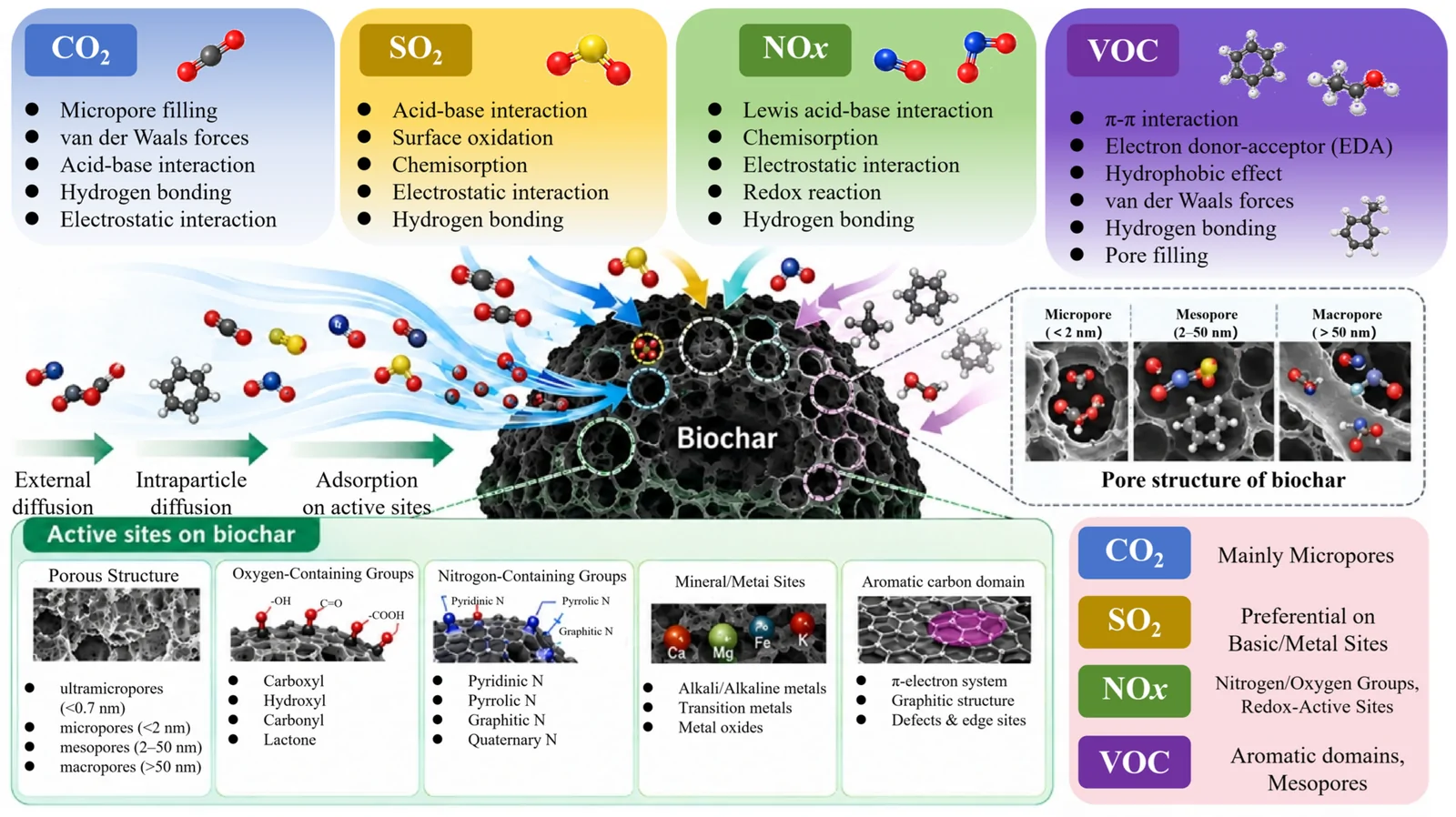
Schematic diagram of the mechanism by which biochar adsorbs polluted gases.

Adsorption is strictly defined as a surface phenomenon involving the transfer of molecules from a fluid phase (a liquid or gas) to the surface of a solid, forming an interfacial layer. This process is typically driven by physical forces (such as van der Waals forces) or chemical bonds (such as covalent or ionic bonds), and is usually reversible. The reverse process is known as desorption. ‘Sorption’, on the other hand, is a broader, umbrella term that encompasses both adsorption (surface enrichment) and absorption (i.e., the entry of a substance into the bulk phase). The process by which adsorbate molecules transfer from the gas phase or liquid phase to the surface of an adsorbent is divided into two basic mechanisms: physical adsorption and chemical adsorption. Physical adsorption refers to the accumulation of adsorbates on the surface or within the pores of an adsorbent via weak intermolecular forces (primarily van der Waals forces), during which no chemical bonds are formed or broken. Chemical adsorption refers to the process whereby strong chemical bonds are formed between the adsorbate and active sites on the adsorbent surface, usually accompanied by electron transfer or atomic rearrangement. Compared with physical adsorption, chemical adsorption is generally irreversible and plays a dominant role in determining adsorption stability. The physical structure of biochar plays a crucial role in gas adsorption performance. During biomass pyrolysis, carbon framework corrosion and volatile release lead to the formation of a hierarchical pore structure, including ultramicropores (<0.7 nm), micropores (<2 nm), mesopores (2–50 nm), and macropores (>50 nm), as illustrated in Figure 7.

The physical structure of biochar is a key factor governing its gas adsorption performance. During biomass pyrolysis, the release of volatile components and the rearrangement of the carbon framework lead to the formation of a hierarchical pore structure, including ultramicropores (<0.7 nm), micropores (<2 nm), mesopores (2–50 nm), and macropores (>50 nm). Macropores (>50 nm) primarily act as transport channels, facilitating rapid diffusion of gas molecules into the interior of biochar particles [111]. Mesopores (2–50 nm) act as intermediate pathways that connect external surfaces with internal active sites, enhancing mass transfer efficiency [112]. Micropores (<2 nm) serve as the principal adsorption sites and dominate adsorption capacity during adsorption–desorption processes [113,114]. Adsorption within micropores typically follows a pore-filling mechanism. According to micropore filling theory, adsorption potential within micropores increases significantly, accelerating the occupation of available pore volume by adsorbate molecules [115]. Consequently, the superior adsorption performance of biochar is strongly associated with its well-developed microporous structure and high specific surface area [116,117]. Gas transport within biochar is governed by pore diffusion, which can be classified into molecular diffusion and Knudsen diffusion [118]. Molecular diffusion dominates when the pore diameter exceeds the mean free path of gas molecules, whereas Knudsen diffusion becomes dominant when the pore size is smaller than the mean free path. For microporous biochar, gas transport is primarily controlled by Knudsen diffusion [119].

However, a single type of biochar cannot achieve high adsorption capacities for all pollutants present in flue gas, or the combined effects of multiple pollutants may result in low adsorption values for any single pollutant. For example, the adsorption of NO is closely associated with micropores (<2 nm) and mesopores (2–50 nm), whereas the adsorption of SO_2_ relies more heavily on a larger specific surface area and a broader mesopore/macropore structure. This disparity makes it difficult for a single biochar material to achieve optimal adsorption performance for multiple pollutants simultaneously, limiting its application in multi-component flue gas environments.

The chemical properties of biochar, including surface functional groups, pH, surface charge, and mineral composition, can enhance its adsorption capacity for pollution gases. To achieve efficient chemical adsorption, it is necessary to introduce or enhance active surface sites through targeted chemical modification. Generally, the modification strategies typically involve treatment with alkaline or metal-containing reagents, such as KOH [51,120], NH_4_OH [26], FeCl_3_ [121], or FeSO_4_ [122]. Surface functional groups can be effectively modulated through chemical oxidation, high-temperature reduction, and related treatments [123]. In particular, oxygen-containing functional groups (such as carboxyl, hydroxyl, and phenolic groups) are crucial for adsorption, as they participate in ion exchange, surface complexation, and redox reactions, enhancing interactions with target pollutants. For example, Fan et al. [124] demonstrated that aging treatment combined with alkaline (NaOH–H_2_O_2_) or acidic (HNO_3_) treatment significantly increased the content of oxygen-containing functional groups on the biochar surface within a temperature range of 20–800 °C. Although these treatments resulted in mass losses of approximately 14% (alkaline) and 30–33% (acidic), the cadmium adsorption capacity increased by up to 21.2%, highlighting the key role of surface chemistry in adsorption performance.

Nitrogen-containing functional groups play a crucial role in enhancing the chemisorption of acidic gases such as CO_2_, SO_2_, and NO*x* (particularly NO_2_). This enhancement is primarily attributed to the electronic characteristics of nitrogen atoms and the increased surface polarity of biochar. As nitrogen atoms possess a lone pair of electrons, they behave as Lewis bases and are capable of forming coordinate bonds with gaseous molecules that act as Lewis acids, producing a stronger adsorption force than that resulting from physical adsorption, which is primarily governed by van der Waals forces. [125]. During pyrolysis, especially at temperatures above 400 °C, the aromatic carbon framework remains relatively stable, allowing nitrogen functionalities (e.g., pyridinic nitrogen) to be retained within the carbon matrix [126]. These nitrogen species not only increase surface polarity but also significantly enhance CO_2_ adsorption capacity [127]. Furthermore, optimization of nitrogen doping can simultaneously regulate pore structure and surface chemistry, maximizing adsorption performance [128]. However, excessive introduction of nitrogen-containing species, particularly via direct impregnation, may block pores and reduce the number of accessible adsorption sites. Consequently, in situ nitrogen self-doping through pyrolysis of nitrogen-rich biomass has emerged as a preferred strategy, because of the enhancement of the nitrogen functionality and the preserving pore accessibility.

The pH of biochar changes following chemical modification. The pH of biochar is another critical parameter that influences adsorption behavior by regulating both surface charge and functional group speciation. It also determines the chemical form of pollutants (e.g., ionization states), governing their interactions with the biochar surface. In general, acidic biochar favors the adsorption of cationic species, whereas alkaline biochar is more effective for acidic gases. For instance, Xu et al. [18] reported that pig manure-derived biochar, despite possessing the lowest specific surface area, exhibited the highest CO_2_ adsorption capacity (23 mg/g), which was primarily attributed to its strong alkalinity (pH = 10.4), outperforming biochars derived from wheat straw and sewage sludge.

### 3.1. The Sorption of CO_2_

Micropore filling is the dominant mechanism governing CO_2_ physisorption, particularly for pore diameters of 0.3–0.8 nm, with pores smaller than 0.5 nm exhibiting superior adsorption performance [129]. Various activation strategies, including ball milling, CO_2_ activation, and steam activation, have been demonstrated to effectively regulate pore structure and enhance CO_2_ adsorption capacity. Ball milling reduces particle size, increases surface area, and exposes additional adsorption sites. For example, Zhang et al. [2] reported that ball-milled biochar exhibited significantly enhanced CO_2_ adsorption capacities compared with pristine biochar, corresponding to increases of 23.52%, 9.50%, and 8.93% in comparison to untreated biochar. CO_2_ and steam activation further improve adsorption performance by increasing pore volume and surface area [130].

Temperature and humidity exert significant influences on CO_2_ adsorption. Elevated temperatures enhance diffusion kinetics but weaken van der Waals interactions, reducing adsorption capacity. From a thermodynamic perspective, the adsorption of CO_2_ by biochar is generally a spontaneous and exothermic process. As the temperature gradually rises, the thermal motion of molecules causes the adsorption capacity of biochar to increase. According to Le Châtelier’s principle and the van’t Hoff equation, for exothermic reactions or processes, excessively high temperature causes the equilibrium to shift towards desorption, thereby reducing the equilibrium adsorption capacity [131]. For unmodified biochar, the adsorption mechanism relies primarily on physical interactions such as microporous filling and van der Waals forces, with the heat of adsorption typically ranging from 20 to 40 kJ/mol [132]. Water vapor competes with CO_2_ for adsorption sites and may block micropores, significantly decreasing adsorption efficiency. Under a CO_2_ partial pressure of 0.15 bar, the CO_2_ loading capacity of the optimized nitrogen-doped biochar at 30% relative humidity can reach 0.5–1.5 mmol/g of dry CO_2_ increment. This synergistic effect stems from the participation of water molecules in the CO_2_ hydration reaction, which reduces the activation energy required to form bicarbonate intermediates, enhancing the chemical adsorption of the biochar. However, when the relative humidity exceeds 60%, excess water molecules undergo capillary coalescence within the pores, leading to the swelling or even loss of amine sites, whilst simultaneously causing severe pore blockage. At this point, the synergistic effect transforms into competitive inhibition, and the CO_2_ adsorption capacity drops to a level below that observed in the dry state [133]. Therefore, optimizing operating conditions requires balancing temperature and humidity to achieve optimal adsorption performance [134].

Enhancing biochar basicity via alkaline treatment, nitrogen functionalization, or metal incorporation improves CO_2_ adsorption through acid–base interactions. Greater surface basicity suppresses acidic oxygen-containing groups (e.g., carboxyl and phenolic hydroxyl groups), reducing repulsion with CO_2_ and increasing selectivity. This effect is especially useful in humid conditions, where basic surfaces are more inclined to adsorb CO_2_ rather than water molecules [135]. Luo et al. [136] modified Enteromorpha prolifera-derived biochar using microwave-assisted KOH activation, optimizing pore structure and significantly increasing surface area and heteroatom content (N, O). Response Surface Methodology was used to optimize activation conditions, which further boosted performance. DFT calculations showed that nitrogen and oxygen heteroatoms synergistically enhance surface polarity and electron-transfer interactions with CO_2_, outperforming single-element doping.

Nitrogen functional groups are widely recognized as key contributors to CO_2_ adsorption [100]. Nitrogen doping can promote the development of the pore structure in biochar, increase its specific surface area, provide more space for physical adsorption, and produce a synergistic effect with chemical adsorption. [128,137]. Common nitrogen functionalities include pyridone, pyridinic N, amines, quaternary ammonium, pyridine-N-oxide, nitriles, and pyrrolic N. Among these, pyridone, pyridinic, and pyrrolic groups play dominant roles in CO_2_ adsorption. Pyridinic nitrogen, located within aromatic rings, possesses lone-pair electrons that do not participate in conjugation, resulting in strong basicity and enabling the formation of coordination bonds with CO_2_. It can also facilitate the transition from physisorption to chemisorption by lowering the activation energy barrier. Amino groups (–NH_2_), due to their highly accessible lone pairs, can interact with CO_2_ via protonation or direct coordination [127]. Although pyrrolic nitrogen is less basic due to electron delocalization within the aromatic system, it can still interact with CO_2_ through π-electron interactions. XPS analysis indicates that pyridone groups exhibit the highest adsorption energy, mainly due to hydrogen bonding between hydroxyl-associated hydrogen atoms and electronegative oxygen atoms in CO_2_, while pyridinic groups dominate Lewis acid–base interactions [138].

Nitrogen functionalities increase surface polarity, facilitating the adsorption of CO_2_, which possesses a significant quadrupole moment. The negative electrostatic potential region arising from the lone-pair electrons of the nitrogen atom is complementary to the positive potential centered at the carbon atom of CO_2_. Such quadrupole–quadrupole (or quadrupole–dipole) interactions substantially enhance the binding energy of CO_2_. From a Lewis acid–base perspective, nitrogen sites act as electron donors, while the carbon atom in CO_2_ serves as an electron acceptor, forming coordination interactions that enhance adsorption [129]. To avoid pore blockage associated with post-synthetic amine impregnation, nitrogen-rich biomass precursors can be employed for in situ self-doping. For example, Du et al. [19] utilized nitrogen-rich pomelo peel as a precursor and ZnCl_2_ as an activating agent to produce porous carbon without additional nitrogen sources. The resulting material exhibited high CO_2_ adsorption capacities of 3.01 mmol/g at 25 °C and 1 bar, and 4.83 mmol/g at 0 °C and 1 bar.

Zhang et al. [137] developed an activation-free strategy by co-carbonizing biochar with a zeolitic imidazolate framework (ZIF-8) to produce nitrogen-doped porous carbon. The introduction of nitrogen functionalities enhanced the synergistic interactions between oxygen- and nitrogen-containing groups, particularly in the presence of C–O and C=O groups associated with graphitic nitrogen. As a result, adsorption energies increased significantly (from 13.67 to 16.42 kJ/mol for aryl–C–O–gN and from 16.18 to 21.46 kJ/mol for aryl–C=O–gN). DFT calculations further confirmed that the synergistic effect between oxygen functionalities and graphitic nitrogen dominates CO_2_ adsorption, with optimal pore sizes in the range of 0.62–0.72 nm. Feng et al. [139] prepared activated biochar from corn straw via one-step and two-step pyrolysis, followed by H_2_O and NH_3_ ⋅ H_2_O activation. The one-step method favored the formation of functional groups, whereas the two-step method enhanced aromatization. During NH_3_·H_2_O activation, competition between NH_3_ and H_2_O was observed, with NH_3_ suppressing H_2_O gasification and reducing oxygen-containing group formation. For CO_2_ adsorption, nitrogen functionalities contributed more significantly than oxygen-containing groups. Active adsorption sites were primarily located in micropores, while mesopores served as diffusion pathways. Notably, within micropores, functional groups contributed more significantly than pore structure alone.

The aromatic structure of biochar can promote the physical adsorption of CO_2_ through van der Waals interactions, providing an additional driving force for the overall adsorption process. Oxygen-containing functional groups play a dual role in CO_2_ adsorption [140]. Common oxygen functionalities include carboxylic, phenolic, lactonic, and hydroxyl groups [141]. On the one hand, these groups can interact with CO_2_ through hydrogen bonding and other intermolecular forces. For instance, the introduction of carboxyl groups can strengthen van der Waals interactions between the carbon surface and CO_2_, whereas hydroxyl groups are conducive to the formation of hydrogen bonds with CO_2_ molecules. These interactions stabilize the adsorption configuration of biochar with respect to carbon dioxide [142]. Most oxygen-containing functional groups are inherently acidic, and an excessive abundance of acidic sites may hinder the adsorption of the acidic gas CO_2_. Moreover, excessive oxygen functionalization may alter the surface polarity and adsorption behavior of biochar under humid conditions, weakening its CO_2_ capture performance. Because CO_2_ is a nonpolar molecule, its interaction with polar sites is relatively weak. In contrast, water molecules possess stronger local polarity and can preferentially occupy these sites, thus reducing the number of surface sites available for CO_2_ adsorption. In addition, van der Waals interactions between water and CO_2_ may further inhibit CO_2_ uptake, decreasing co-adsorption energy [142].

**Figure 8:**
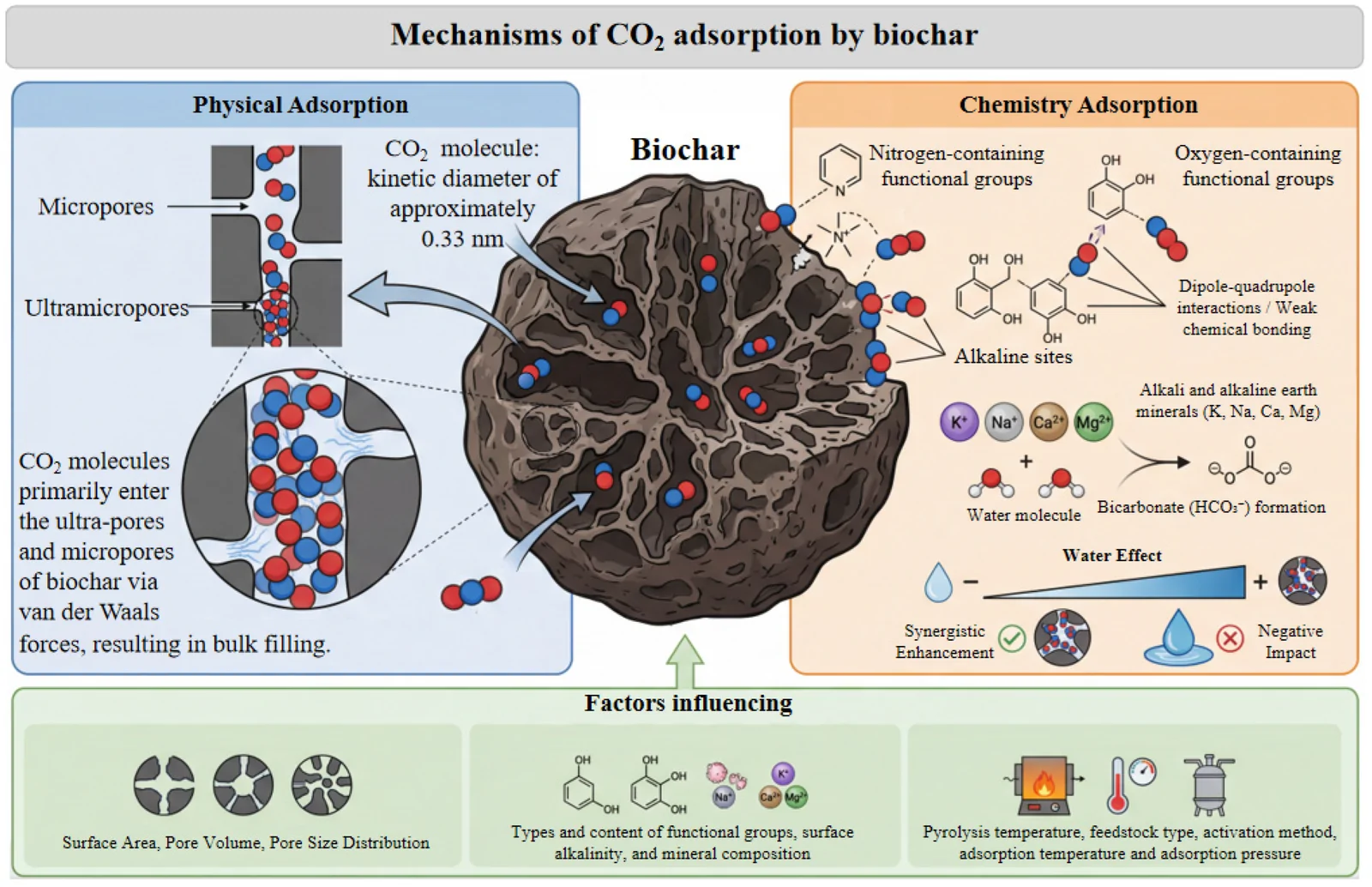
Mechanisms of CO_2_ adsorption by biochar.

Using multiple modification strategies does not always improve CO_2_ adsorption. Igalavithana et al. [143] found that biochar from food and wood waste, activated first with KOH and then with CO_2_, had lower CO_2_ uptake than biochar activated by KOH alone. KOH activation develops the microporous structure, increasing surface area and micropore volume. These two parameters are both critical for the adsorption of CO_2_. However, subsequent CO_2_ activation may lead to excessive activation of biochar, widening some micropores into mesopores (2–4 nm). At low pressure (0–1 bar) and 25 °C, CO_2_ adsorption primarily occurs in ultramicropores smaller than 0.8 nm, while mesopores contribute less. Thus, the reduced adsorption performance after CO_2_ activation is mainly due to the loss of the ultramicroporous structure and the associated reduction in effective surface area. Figure 8 illustrates the mechanism by which biochar adsorbs carbon dioxide.

Compared with other typical solid CO_2_ adsorbents, biochar with a low nitrogen content primarily relies on its physical properties to achieve CO_2_ adsorption, transportation, and storage. Its physical adsorption of CO_2_ remains the primary means of CO_2_ capture. Typically, the microporous structure provides ample anchoring sites for the functional groups introduced through chemical modification, offering a site for the initial capture and enrichment of CO_2_, while the basic functional groups distributed on the surface or within the pores interact more strongly with CO_2_, enhancing the overall adsorption capacity and selectivity. At present, the most effective method for pore formation is modification using KOH; however, following modification, the material usually needs to be rinsed with an acidic solution to render it neutral. Otherwise, its strongly alkaline nature may corrode plant equipment, causing secondary pollution and increasing operating costs.

### 3.2. The Sorption of SO_2_

The kinetic diameter of SO_2_ (~0.36 nm) is well matched to the size of biochar micropores (<2 nm), enhancing van der Waals forces, and increasing adsorption capacity [144]. Studies have found a significant linear correlation between SO_2_ adsorption capacity and micropore volume [66,145]. For example, Zhang et al. [144] demonstrated that both physical activation with carbon dioxide and high-temperature ammoniation can increase the specific surface area of biochar, increasing the adsorption capacity of SO_2_. Excessively high pyrolysis temperatures during the pyrolysis process can lead to an increase in the ash content of biochar, which may block some of the pores; at the same time, they can also destroy the active functional groups on the surface of the biochar, reducing its adsorption capacity.

Physisorption is typically an exothermic process. Thus, elevated temperatures (>100 °C) generally reduce adsorption capacity. In contrast, chemisorption may exhibit different temperature dependence. For amine-modified biochar, adsorption capacity increases with temperature from 50 to 100 °C, with chemisorption becoming dominant at approximately 100 °C [14,146].

Humidity also plays a critical role in SO_2_ adsorption. Moderate humidity levels (15–60%) can promote the formation of a thin water film on the biochar surface, facilitating SO_2_ dissolution and subsequent oxidation (e.g., SO_2_ → H_2_SO_4_), thereby enhancing adsorption capacity [147]. However, excessive humidity (>60%) may lead to competitive adsorption between water vapor and SO_2_ for active sites, reducing adsorption efficiency.

The introduction of nitrogen-containing functional groups, such as –C–N and –NH_2_, can markedly improve the SO_2_ adsorption performance of biochar. These nitrogen species act as Lewis bases and enhance the chemisorption of acidic SO_2_ via acid–base interactions. Nitrogen-doped biochar has therefore been widely regarded as a renewable, cost-effective, and promising adsorbent for SO_2_ removal [145]. Shao et al. [66] improved the physicochemical properties of corncob-derived biochar through CO_2_ activation followed by methyldiethanolamine (MDEA) impregnation. After CO_2_ activation, the specific surface area increased from 56.91 to 755.35 m^2^/g, whereas subsequent MDEA impregnation reduced it sharply to 25.54 m^2^/g or even lower. Nevertheless, after impregnation with 10% MDEA, the maximum SO_2_ adsorption capacity increased from 57.8 to 156.2 mg/g. These results indicate that, at 393 K, surface nitrogen functionalities play a more decisive role than pore structure in SO_2_ adsorption, with quaternary nitrogen being the dominant active species. Zhang et al. [146] employed a high-temperature CO_2_–NH_3_ modification strategy to enhance SO_2_ adsorption. The results of in situ DRIFTS based on 2D perturbation correlation method show that the amino groups, unsaturated bonds, and the hydrogen bonds associated with dimeric, π-type, polymeric, chelation, and bridge structures on nitrogen-doped biochar exhibited SO_2_ adsorption ability. SO_2_ reacted with the biochar surface to generate the S-H, thiocyanato/isothiocyanato, ammonium/thiourea, SO_4_^2−^/sulphate R-O-SO_2_-OR’, S = O, and SO_3_^2−^ at 30 °C. These formation reactions improved, and the S-H was converted to other sulfide groups as the adsorption temperature increased to 60 °C. At a high adsorption temperature (120 °C), the stable SO_4_^2−^/sulphate R-O-SO_2_-OR’ would be formed.

**Figure 9:**
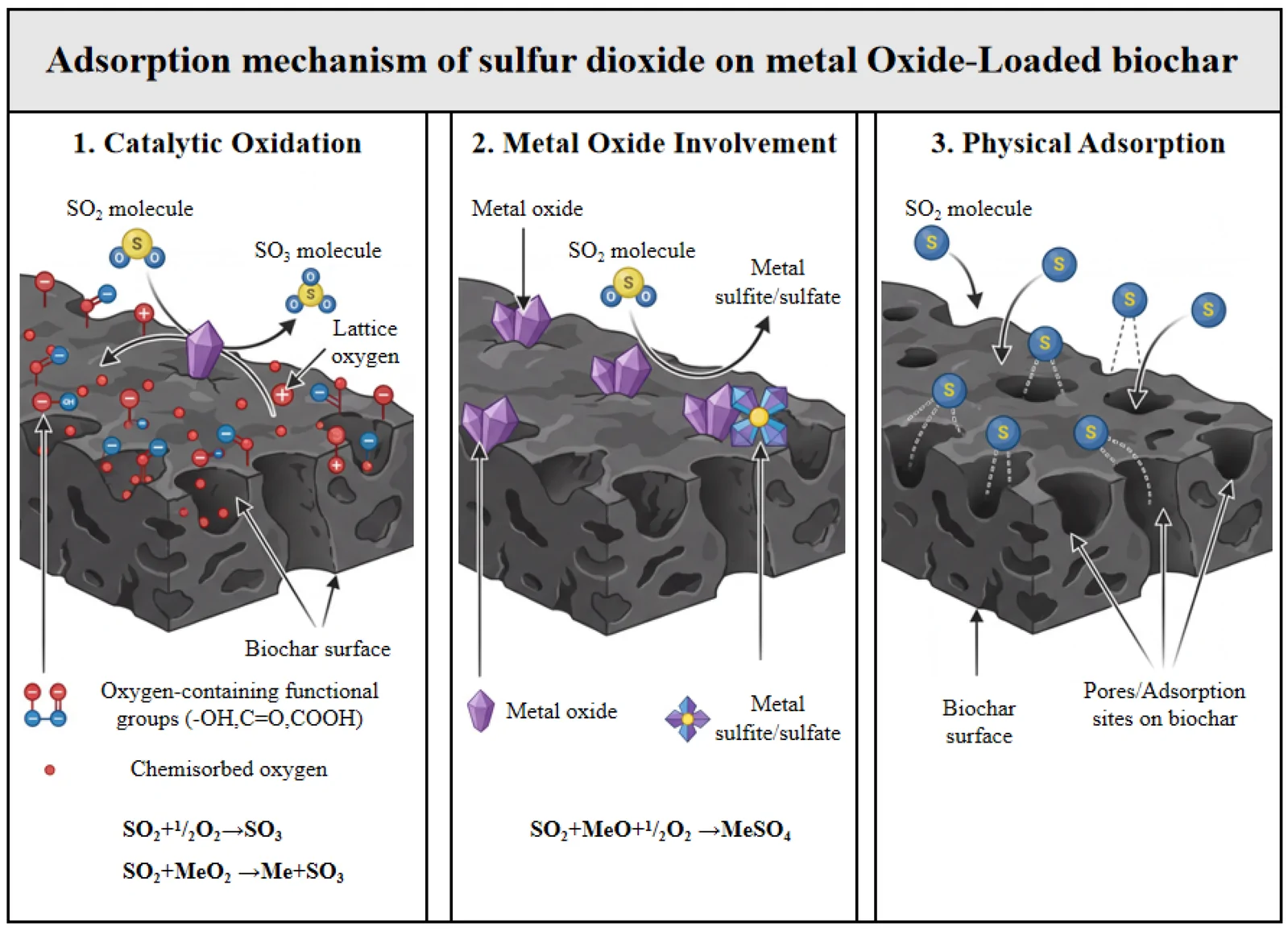
Mechanism of sulfur dioxide adsorption by metal oxide-supported biochar.

Loading metal oxides onto biochar can enhance sulfur dioxide capture capacity. When metals are dispersed on high-surface-area porous carbon supports, the pore network facilitates uniform distribution of metal oxides while suppressing particle agglomeration. Cu-modified biochar has been reported to exhibit strong SO_2_ adsorption capacity [27], as shown in Figure 9, with SO_2_ ultimately being converted into stable copper sulfate species. This not only improves SO_2_ removal efficiency but also stabilizes the adsorption products.

(19) $$\mathrm{CuO}+SO_{2}+\frac{1}{2}O_{2}\to {\mathrm{CuSO}}_{4}$$

In addition to introduced metal species, naturally occurring minerals in biochar, especially Ca and Ce, can also contribute substantially to SO_2_ removal by directly reacting with SO_2_ to form stable sulfate compounds such as CaSO_4_·2H_2_O. The contribution of these mineral phases to total SO_2_ uptake can range from 44.6% to 85.5%. Under high-temperature carbonization conditions (600–950 °C), more reactive Ca-containing oxides are formed, which play a crucial role in SO_2_ capture. Xu et al. [148] prepared biochars from dairy manure, rice husk, and sewage sludge, all of which showed considerable potential for SO_2_ adsorption.

SO_2_(gas) ↔ SO_2_(ads) (20)

(21) $$SO_{2}(\mathrm{ads})+H_{2}O↔H_{2}SO_{3}^{*}↔2H^{*}+SO_{3}^{2-}$$

(22) $$2SO_{2}(\mathrm{ads})+{2H}_{2}O+O_{2}↔2SO_{4}^{2-}+4H^{+}$$

2H^+^ + CaCO_3_ → Ca^2+^ + CO_2_ ↑ + H_2_O (23)

(24) $$2SO_{3}^{2-}+3Ca^{2+}+SO_{4}^{2-}+12H_{2}O\to Ca_{3}(SO_{3}{)}_{2}SO_{4}\cdot 12H_{2}O↓$$

(25) $$Ca^{2+}+SO_{4}^{2-}+2H_{2}O\to \mathrm{CaS}O_{4}\cdot 2H_{2}O↓$$

(26) $$Fe_{2}O_{3}+4SO_{4}^{2-}+8H^{+}\to Fe_{2}(SO_{4}{)}_{3}\cdot H_{2}SO_{4}\cdot 2H_{2}O$$

**Figure 10:**
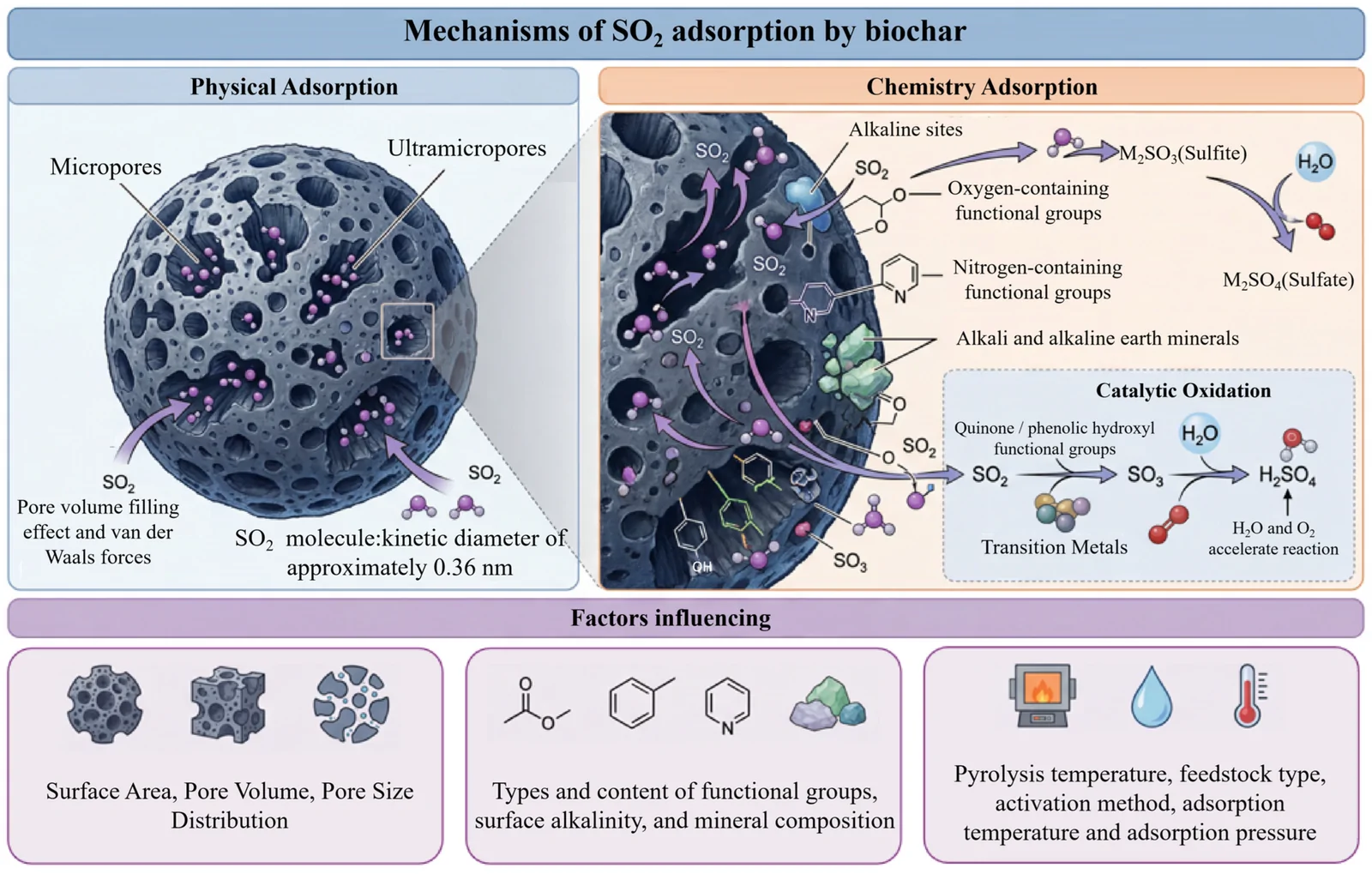
Mechanisms of SO_2_ adsorption by biochar.

The presence of moisture and oxygen can further promote SO_2_ adsorption and transformation on biochar. Mineral components in biochar may react with adsorbed SO_2_ to form various sulfur-containing mineral phases, enhancing SO_2_ retention. In practical flue gas systems, the coexistence of other gaseous components also affects the adsorption behavior of SO_2_. For instance, O_2_ can promote the oxidation of SO_2_ to SO_3_, strengthening chemisorption, whereas NO may exert a synergistic effect by forming intermediates, which significantly enhance SO_2_ uptake [14]. During the carbothermal reaction between SO_2_ and carbon, extensive sulfur doping and the formation of thiophene and sulfur oxide functionalities may occur. Dynamic adsorption tests have shown that these sulfur-containing groups promote SO_2_ removal by altering the surface charge distribution of carbon, strengthening physisorption of SO_2_ while also enhancing chemisorption at unsaturated carbon sites [149]. These complex, multi-component adsorption behaviors require further experimental and computational modeling to understand and optimize the application of biochar under actual flue gas conditions. Consistent with this, Zhang et al. [14] reported that pristine biochar exhibited an SO_2_ adsorption capacity of 78.9 mg/g, whereas nitrogen-doped biochar reached 135 mg/g in a mixed SO_2_/NO/CO_2_/O_2_ system. This enhancement was attributed to the formation of nitrogen–sulfur compounds via interactions among SO_2_, NO, and oxygen radicals in the presence of O_2_, while oxygen-containing surface groups provided additional oxygenated active sites, further promoting chemisorption. Figure 10 illustrates the mechanism by which biochar adsorbs sulphur dioxide.

### 3.3. The Sorption of NOx

The molecular properties of NO and NO_2_ determine the differences in their initial adsorption behavior. NO is a neutral open-shell radical with extremely low polarity and weak oxidizing power. Its adsorption on non-functionalised carbon surfaces relies primarily on weak van der Waals forces or π–π interactions, exhibiting typical characteristics of physical adsorption. Due to its lack of strong polarity, the interaction between NO and the inert graphitised planes of biochar is extremely weak, and the heat of adsorption is low, so desorption occurs very readily upon heating. Typically, NO must first be catalytically oxidized at active sites on the biochar surface (such as transition metals Fe, Cu, and Mn or quinone-type oxygen-containing functional groups) to form NO_2_ or higher-valent nitrogen oxides before it can be effectively fixed. In the absence of active sites, NO exhibits only transient physical adsorption, and the breakthrough curve rapidly reaches saturation. Furthermore, nitrogen-doped biochar can enhance its electron-donating capacity towards NO by introducing active sites such as pyridine nitrogen or pyrrole nitrogen, thereby promoting its activation and subsequent reduction reactions. In contrast, NO_2_ possesses higher polarity and strong oxidizing properties and readily dimerises to form N_2_O_4_. This makes it more prone to strong chemical adsorption onto the biochar surface via chemical bonding. The main pathways include: (1) Acid–base neutralization reactions with surface basic sites (such as ash components like K, Ca, and Mg, or added alkali metals), resulting in the formation of stable surface nitrites and nitrates. (2) Addition or substitution reactions at oxygen-containing functional groups, forming surface-bound -ONO or -NO_2_ groups. (3) Surface disproportionation reactions, yielding NO and surface nitrates. As a strong electron acceptor, NO_2_ is capable of undergoing significant electron transfer with oxygen-containing functional groups or basic sites on the biochar surface, resulting in a highly exothermic adsorption process that exhibits pronounced irreversibility at low temperatures.

The micropores (<2 nm) in biochar offer a large specific surface area and strong adsorption potential for NO_2_, while the mesopores (2–50 nm) facilitate the diffusion of NO molecules into the micropore region, enhancing overall adsorption efficiency [150,151]. Good pore connectivity prevents premature saturation of surface sites and ensures that internal active sites are not utilized inefficiently. It is crucial for preventing pore blockage and ensuring the efficient transport of NO_2_ molecules to internal adsorption sites. Verma et al. [152] prepared a composite adsorbent (MLBC) by modifying hemp-stem-derived biochar (BC) with red clay. This modification significantly improved the physical structure of the material. The original biochar had a low specific surface area (6.997 m^2^/g) and total pore volume (0.019 cm^3^/g), resulting in a NO_2_ adsorption capacity of only 0.079 mg/g. In contrast, the modified sample (MLBC-3) exhibited a substantial increase in both specific surface area (90.887 m^2^/g) and pore volume (0.084 cm^3^/g), with its NO_2_ adsorption capacity also significantly improving to 14.65 mg/g.

Adsorption temperature exerts a dual effect on NO*x* removal efficiency. At low temperatures (<100 °C), physisorption is typically favored, as van der Waals interactions dominate the adsorption process. However, as the temperature increases to 100–300 °C, chemisorption and metal-catalyzed oxidation processes may be promoted. Zbair et al. [151] prepared biochar from fresh and waste wood sawdust (denoted as B-WSF and B-WSU, respectively). At 22 °C, both these two samples exhibited a gradual increase in NO_2_ adsorption without detectable NO release, indicating the absence of reduction reactions. This suggests that NO_2_ adsorption at low temperatures is primarily governed by physisorption. In contrast, with increasing temperature, the adsorption process was accompanied by NO formation, indicating the occurrence of chemical adsorption and reduction reactions in which NO_2_ was partially reduced to NO. Meanwhile, the adsorption capacity increased significantly with temperature. Further enhanced oxidation of surface functional groups and the formation of nitrogen-containing species were revealed by XPS analysis, which confirmed that chemisorption and surface reactions are promoted by elevated temperatures. Li et al. [153] investigated NO*x* adsorption under various atmospheres in the temperature range of −20 to 30 °C. As the temperature decreased from 30 °C to −20 °C, the NO*x* adsorption capacity increased by 4.61 times. The proportion of physically adsorbed NO*x* increased from 2.9% to 45.5%, while the contribution of NO_2_ to total physisorption increased from 0 to 71.6%. These results demonstrate that lower temperatures are conducive to the oxidation of NO*x* and the adsorption of NO_2_ on carbon-based materials.

Acidic NO*x* species can undergo acid–base neutralization reactions with basic sites on the biochar surface, constituting a typical chemisorption process. This mechanism is particularly effective for NO_2_, which is more acidic and reactive than NO, and is analogous to the adsorption behavior of other acidic gases such as SO_2_ and CO_2_. Because NO is relatively inert, it is difficult to capture efficiently through simple physisorption alone. Therefore, nitrogen-containing functional groups are critical for improving NO adsorption performance [154,155]. NO_2_, owing to its strong polarity and partial negative charge, readily interacts with basic surface sites through acid–base reactions, making chemisorption the dominant mechanism. Although this behavior is generally similar to that of SO_2_ and CO_2_, NO_2_ exhibits a stronger adsorption affinity due to its higher reactivity and larger dipole moment. Nitrogen sites located at the edges of the biochar structure are considered key active centers for the reduction of NO to N_2_, while oxygen-containing surface groups significantly influence the reactivity of these nitrogen sites [156]. Nitrogen functionalities such as pyridinic N, pyrrolic N, and quaternary N can activate NO molecules through coordination interactions between their lone-pair electrons and the vacant orbitals of NO [155]. This activation may promote the oxidation of NO into the more readily adsorbed NO_2_, indirectly enhancing overall NO*x* removal efficiency.

Although oxygen-containing functional groups are generally acidic in nature, such as carboxyl (–COOH) and hydroxyl (–OH), they can still assist NO_2_ adsorption through hydrogen bonding because of the high polarity of NO_2_. However, when oxygenated groups act alone as the dominant adsorption sites, their overall contribution remains limited compared with that of basic nitrogen functionalities. Yang et al. [154] prepared pristine and modified biochars from cedar (G-type lignin), elm (G–S type lignin), and bamboo (G–S–H type lignin) via acid washing, nitrogen doping, and combined treatments. The results showed that both acid washing and nitrogen doping improved the NO removal performance of elm-derived biochar, which originally possessed fewer structural defects. Moreover, the affinity of ash elements for oxygen was found to affect nitrogen-doping efficiency and NO removal performance.

Since NO_2_ is a polar molecule that can carry a partial negative charge, positively charged biochar surfaces generated through nitrogen doping or metal ion loading can significantly enhance NO_2_ capture via electrostatic attraction. This electrostatic contribution becomes particularly important in humid environments, where water molecules can intensify polar interactions, promoting NO_2_ adsorption. However, pristine biochar generally carries a negative surface charge because of its inherent oxygen-containing groups. Therefore, to optimize NO_2_ adsorption, the surface charge of biochar should ideally be adjusted to a weakly positive state through targeted modification [157]. Loading positively charged metal ions is also an effective strategy. DFT calculations have further clarified the adsorption and reduction mechanisms of NO_2_ on K-doped biochar, highlighting the decisive role of active sites in controlling the reaction pathway [158].

### 3.4. The Sorption of VOCs

Micropores serve as the primary adsorption sites for VOCs, with both micropore volume and specific surface area showing a strong positive correlation with adsorption capacity [159]. In particular, narrow micropores (0.33–0.63 nm) are critical for the adsorption of small VOC molecules, such as toluene and acetone. This behavior is attributed to the enhanced adsorption potential within confined micropores, which facilitates efficient capture of small molecules. In addition to micropores, mesopores and macropores play essential roles as transport pathways, promoting the diffusion of VOCs into microporous regions and improving overall adsorption kinetics [160]. This synergistic effect is especially pronounced in hierarchical porous structures, which integrate micro-, meso-, and macropores to simultaneously optimize adsorption capacity and mass transfer efficiency. Feng et al. [159] prepared a series of biochars from corn straw via chemical activation with KOH and ZnCl_2_. The sample of 200NH-CS-KOH-800C exhibited a micropore volume of 0.95 cm^3^/g, accounting for 84.10% of the total pore volume. The micropore specific surface area was 1834.41 m^2^/g (92.52% of total surface area). Correspondingly, this sample achieved a phenol adsorption capacity of 93.50 mg/g, which was significantly higher than that of the poorly developed sample 200NH-CS-800C (micropore volume: 0.10 cm^3^/g; adsorption capacity: 43.31 mg/g).

Ball milling can increase the number of accessible adsorption sites for VOCs [161]. Further modification using oxidizing or alkaline agents (e.g., H_2_O_2_ or NH_4_OH) can significantly enhance physicochemical properties. For instance, H_2_O_2_ treatment can increase the specific surface area of ball-milled biochar by an additional increase of 1.23 times [26]. Xiang et al. [161] prepared biochar from hickory wood, achieving a specific surface area of 221.5 m^2^/g at 600 °C, which further increased to 304.84 m^2^/g after ball milling (a 1.4-fold enhancement). Correspondingly, the adsorption capacities of the ball-milled biochar (HWBM600) for acetone, ethanol, and toluene increased by 1.2–1.5 times, highlighting the critical role of surface area in VOCs adsorption. Zhuang et al. [162] reported that ball milling significantly increased the specific surface area of biochar derived from rice husk, corn straw, and pine sawdust. A strong linear correlation (R^2^ > 0.8895) was observed between toluene adsorption capacity and specific surface area, confirming that physisorption is the dominant mechanism. The increased microporosity not only provides additional adsorption sites but also shortens diffusion pathways, facilitating rapid transport of toluene molecules into internal pores where they are effectively retained.

Oxygen-containing functional groups on biochar surfaces, such as carboxyl (–COOH) and phenolic hydroxyl (–OH), can enhance surface polarity and hydrophilicity, promoting the adsorption of polar VOCs (acetone and ethanol) through dipole–dipole interactions and hydrogen bonding [26]. Most oxygen-containing groups gradually decompose during high-temperature pyrolysis. Consequently, biochar produced at relatively low pyrolysis temperatures (250–450 °C) usually contains more organic functional groups and incompletely carbonized organic residues.

Carbonyl groups (C=O), as important oxygen-containing functionalities, can act as electron acceptors and promote the oxidative adsorption of VOCs, such as toluene, via electron-transfer mechanisms. The presence of carbonyl groups, enhances the affinity of biochar toward aromatic compounds containing a group capable of undergoing electronic substitution [58]. Recent studies have systematically reviewed the formation mechanisms, characterization methods, engineering applications, and pollutant-removal functions of oxygen-containing groups, further emphasizing their critical role in biochar functionalization [160]. The aromaticity and hydrophobicity of biochar are also key determinants of VOCs adsorption performance. Biochars with higher aromaticity, typically indicated by lower H/C ratios, can effectively adsorb aromatic VOCs such as toluene and chlorobenzene through π–π interactions. This effect becomes more pronounced at high pyrolysis temperatures (600–900 °C), which increase the degree of graphitization and thus strengthens π-electron interactions [162]. In parallel, hydrophobicity generally increases with pyrolysis temperature, helping suppress competitive adsorption by water molecules and improving the adsorption efficiency for nonpolar VOCs such as cyclohexane [12].

Pyrolysis temperature exerts a decisive influence on both pore structure and surface chemistry. In general, the specific surface area of biochar increases with increasing pyrolysis temperature [12]. High-temperature pyrolysis (600–900 °C) significantly enhances microporosity, specific surface area, and hydrophobicity. Because of the strong affinity between the adsorbent and nonpolar molecules through π–π interactions, all of these species are essential for the adsorption of nonpolar VOCs such as benzene and cyclohexane [161]. In addition, microwave-assisted pyrolysis has emerged as a rapid and effective preparation method that can control both the heating rate during carbonization and the activation temperature, increasing the specific surface area. For example, trace FeCl_3_ addition can promote the formation of micropores, improving the adsorption of aromatic VOCs [163].

Functionalization strategies further expand biochar’s potential for VOCs adsorption. Nitrogen doping is an important route for introducing nitrogen-containing groups, such as pyridinic and pyrrolic nitrogen, via ammonia treatment and urea modification. These nitrogen species can alter the electronic structure and surface chemistry of biochar, enhancing the adsorption of aromatic VOCs, such as toluene, through polar interactions and π–π conjugation. Pyrrolic nitrogen, in particular, can improve adsorption selectivity through electrostatic interactions. Metal loading is another effective approach. Transition metals (Cu, Mn, and metal–organic frameworks such as Cu-BTC) can be incorporated onto biochar surfaces to exploit metal–VOCs coordination and catalytic oxidation, improving adsorption performance at elevated temperatures. In addition, plasma and microwave treatments have attracted increasing attention. Plasma treatment (N_2_ or O_2_ plasma) can introduce nitrogen-containing groups and oxygen-containing groups while optimizing pore structure, whereas microwave treatment promotes micropore development and a more uniform distribution of functional groups, enhancing VOCs adsorption efficiency [163].

Zhang et al. [85] demonstrated that an optimized carbon model showed good agreement with the pore-structure parameters of nitrogen-doped biochar, with a fitting degree of over 80% for toluene adsorption capacity at room temperature. The adsorption capacities of nitrogen-doped biochar for different VOCs ranged from 184 to 317 mg/g, following the order phenol > naphthalene > p-xylene > toluene ≈ benzene ≈ chlorobenzene under single-component adsorption conditions. Among these compounds, phenol exhibited the highest adsorption due to its greater polarity, which enabled stronger electrostatic interactions with the adsorbent. As the adsorption temperature increased, benzene showed the smallest decline in adsorption capacity because of its relatively low molecular weight. Moreover, increasing gas concentration enhanced van der Waals interactions between VOC molecules and between VOCs and porous carbon, increasing adsorption capacity. Feng et al. [159] prepared chemically activated biochar from corn straw and investigated its adsorption behavior in liquid-phase systems. The results showed that chemical activation constructed abundant O/N-containing functional groups on the pore surface, forming a hierarchical functionalized pore structure that strongly promoted phenol adsorption. Specifically, the mesoporous structure mainly determined phenol uptake, while hydroxyl groups strengthened adsorption through enhanced electrostatic interactions, and N-6 species increased van der Waals forces by modifying adsorption sites, further improving phenol adsorption. Mosleh et al. [164] produced biochar from wheat straw and hardwood via pyrolysis and subsequently modified the materials by impregnation with HCl and NaOH solutions. These treatments significantly increased specific surface area, pore volume, and surface chemical activity. Owing to alkaline hydrolysis, the specific surface area of the raw samples increased markedly after treatment, from 58.38 to 121.72 m^2^/g for wheat straw biochar and from 19.92 to 62.45 m^2^/g for hardwood biochar. Although slight decreases were observed after benzoic acid modification, the treated biochar exhibited enhanced surface chemistry and more oxygen-containing groups, which acted as efficient adsorption sites for toluene and p-xylene. As a result, toluene adsorption increased from 32.5 to 125.3 mg/g for wheat straw biochar and from 27.6 to 83.3 mg/g for hardwood biochar.

### 3.5. Gas-Competitive Adsorption

Biochar, as a low-cost adsorbent with considerable potential, shows great promise for application in the field of flue gas desulfurisation and denitrification; however, its transition from ideal laboratory conditions to actual industrial flue gas environments still faces significant challenges. The composition of flue gas generated from the combustion of coal or biomass is extremely complex, typically comprising sulfur dioxide (SO_2_), nitrogen oxides (NO*x*), carbon dioxide (CO_2_), water vapor (H_2_O), oxygen (O_2_) and particulate matter, with temperatures generally ranging from 100 to 200 °C. Although a large body of existing research has confirmed the excellent adsorption performance of biochar under low-temperature conditions and with single or binary gas components, the mechanisms underlying performance degradation under conditions simulating real flue gas—characterized by multi-component competitive adsorption and high temperature and humidity—still require in-depth analysis.

**Figure 11:**
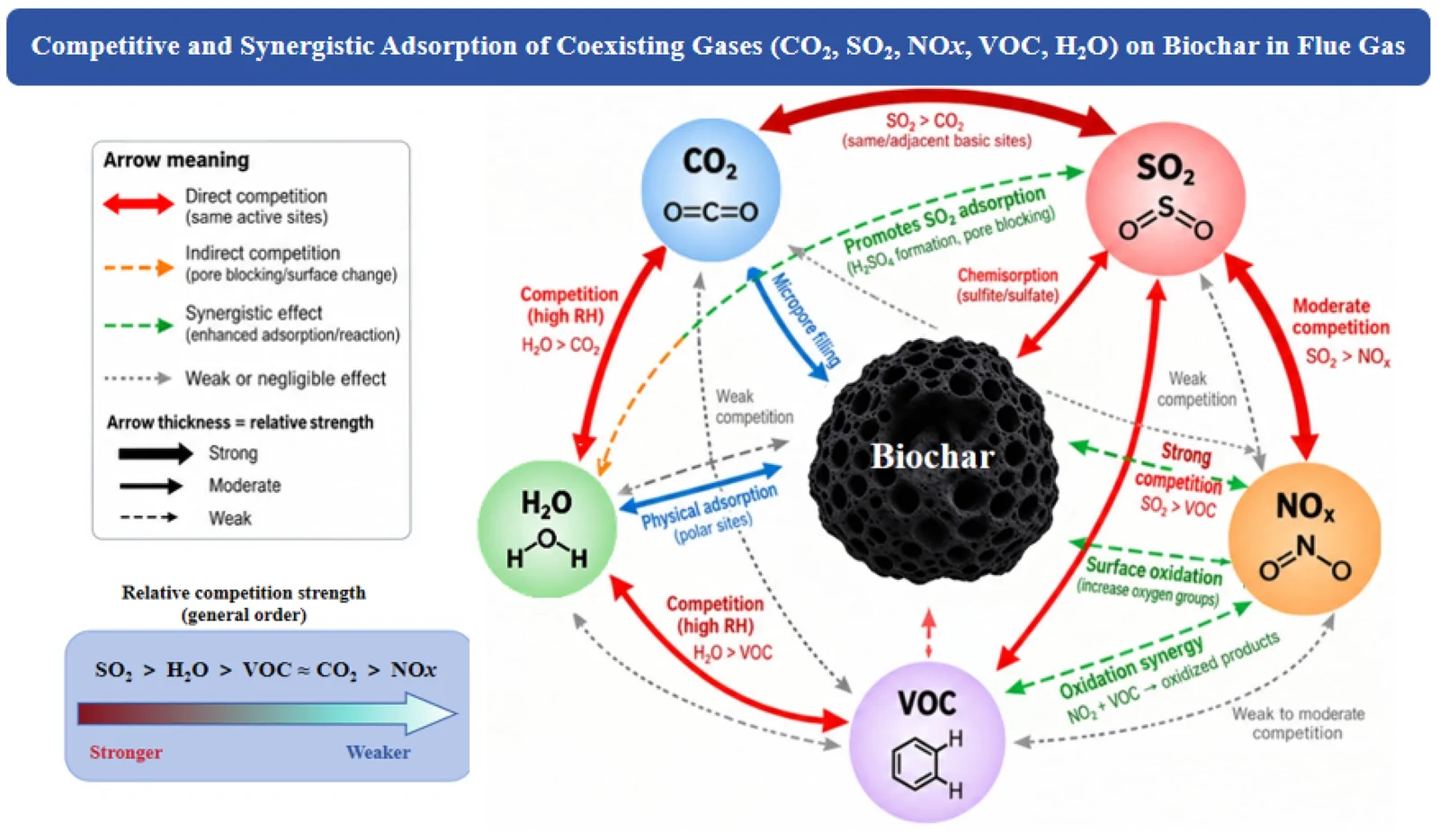
Competitive and synergistic adsorption mechanisms of coexisting flue gas components on biochar.

Figure 11 summarizes the competitive and synergistic interactions among the major flue gas components (CO_2_, SO_2_, NO*x*, VOCs, and H_2_O) during adsorption on biochar. Direct competition occurs when different gas molecules occupy the same adsorption sites, including micropores, basic functional groups, oxygen-containing groups, and π-electron-rich carbon surfaces. Among all components, SO_2_ exhibits the strongest competitive adsorption because of its high polarity and chemisorption affinity toward biochar, allowing it to preferentially occupy active sites and suppress the adsorption of CO_2_, VOCs, and, to a lesser extent, NO*x*. Water vapor also significantly influences adsorption behavior, particularly under high relative humidity, where H_2_O competes with CO_2_ and VOCs for adsorption sites and forms sulfuric acid or sulfate species together with SO_2_, leading to pore blockage and surface modification. These indirect effects further reduce the adsorption capacity of other gases. In contrast, CO_2_ adsorption is dominated by physical adsorption through micropore filling and is therefore more susceptible to displacement by strongly adsorbing species. NO*x* generally exhibits relatively weak competitive adsorption but may oxidize the biochar surface, increasing oxygen-containing functional groups and thereby promoting VOC oxidation or adsorption under certain conditions. Overall, the relative competitive strength of the five gas components generally follows the order: SO_2_ > H_2_O > VOC ≈ CO_2_ > NO*x*. The figure highlights that competitive adsorption in multi-component flue gas is governed not only by direct competition for active sites but also by indirect mechanisms such as pore blockage and surface chemical transformation, emphasizing the importance of considering gas–gas interactions when evaluating biochar performance under realistic flue gas conditions.

## 4. Conventional Approaches for Air Pollutant Removal

Traditional air pollutant control technologies, including adsorption, catalytic combustion, and absorption, have been extensively developed and applied to remove gaseous pollutants. Under appropriate operating conditions, these methods can effectively reduce pollutant emissions. In recent years, biochar has emerged as a highly promising solid adsorbent, and research into its use as a cost-effective and sustainable alternative for the purification of flue gas via adsorption has been increasing. This section first provides a systematic overview of conventional treatment technologies for different categories of gaseous pollutants, followed by a discussion of representative biochar-based adsorption systems. Furthermore, a comparative analysis is presented to evaluate the advantages and limitations of each method.

### 4.1. Adsorption Methods of CO_2_

At present, carbon dioxide capture technologies primarily include amine scrubbing, electrochemical capture, membrane separation, and adsorption. Amine scrubbing is a mature technology widely used for post-combustion carbon dioxide capture, particularly in large stationary sources such as coal-fired power stations [165]. Flue gas containing carbon dioxide enters the absorption tower, where it comes into counter-current contact with a circulating lean amine solution. The carbon dioxide reacts with the amine to form bicarbonate or carbamate compounds, thereby being removed from the gas stream. The CO_2_-rich solution is then conveyed to a desorption tower (regenerator), where heating (typically using steam) releases high-purity CO_2_. The regenerated dilute amine solution is subsequently recirculated back to the absorption tower [166]. Amine scrubbing is one of the most mature and widely applied CO_2_ capture technologies, offering high efficiency and strong selectivity, making it particularly suitable for low-concentration CO_2_ streams [166]. However, the regeneration step requires significant thermal energy resulting in high operating costs. Furthermore, the amine solution is corrosive to equipment, necessitating the use of expensive corrosion-resistant materials. Thermal degradation, oxidative degradation, and solvent volatility may also pose environmental risks [167].

Electrochemical CO_2_ capture is an emerging technology that utilizes redox reactions to enable reversible CO_2_ capture and release under mild conditions [168]. By applying an external voltage, the affinity of redox-active adsorbents or electrolytes toward CO_2_ can be modulated, allowing controllable capture and desorption [169]. Direct electrochemical systems typically generate nucleophilic species via electroreduction in organic solvents, which then react with CO_2_ to form adducts that can later be electrochemically decomposed to release CO_2_. These systems are particularly suitable for low-concentration CO_2_ capture but are limited by high ohmic resistance and sensitivity to water. Indirect electrochemical approaches rely on pH swing or metal–amine complexation mechanisms in aqueous systems, offering lower resistance and improved scalability [170]. However, large-scale deployment remains uncertain, as many systems are still at the proof-of-concept stage. Challenges include high material costs, long-term stability of electrodes and electrolytes, and sensitivity to oxygen in flue gas [171].

Membrane separation technology enables CO_2_ separation via selective permeation, driven by preferential adsorption or molecular sieving [172,173]. Common membrane materials include polymeric, inorganic, and mixed-matrix membranes [174]. Membrane systems are highly flexible and can be scaled to match flue gas flow rate and CO_2_ concentration, making them suitable for applications in power plants and the steel industry. Compared with conventional methods, membrane processes do not involve phase change, resulting in lower energy consumption, compact equipment, and reduced capital and operating costs [175]. Membrane systems are mechanically simple, require minimal maintenance, and avoid the use of chemical solvents, making them environmentally friendly. They can also produce high-purity CO_2_ suitable for industrial applications. Membrane performance can be adversely affected by particulates, sulfur compounds, and moisture in flue gas, which may cause pore blockage or chemical degradation.

Adsorption-based methods can be broadly classified into physisorption and chemisorption [3,176]. Physisorption relies on van der Waals interactions and is typically reversible, with common adsorbents including activated carbon, zeolites, and MOFs [177]. These systems operate at relatively low temperatures (25–100 °C) and exhibit low heats of adsorption (20–40 kJ/mol), resulting in lower regeneration energy requirements. Chemisorption, in contrast, involves the formation of chemical bonds between CO_2_ and functional groups such as amines or metal oxides [178]. Although chemisorption typically offers higher adsorption capacities (>10 mmol/g), it requires higher regeneration temperatures (>300 °C), increasing energy consumption [179]. Adsorption technologies offer high operational flexibility and can accommodate CO_2_ concentrations ranging from 5% to 15%, making them suitable for various industrial applications [180].

Biochar is typically produced via pyrolysis of biomass precursors such as rice husk, wood chips, and agricultural residues [181]. It can also be combined with advanced materials such as MOFs to further enhance CO_2_ adsorption performance [182].To improve adsorption capacity, both physical and chemical activation strategies are commonly employed. Physical activation using steam or CO_2_ can effectively tailor pore structure [69,183], whereas chemical activation with agents such as K_2_CO_3_, KOH, H_3_PO_4_, or urea can significantly increase specific surface area and pore volume [129,136,184,185]. The specific carbon dioxide adsorption property for samples derived from various pretreatment processes are summarized in Table 1.

Biochar, activated carbon, zeolites, and metal–organic frameworks (MOFs) exhibit significant differences in terms of adsorption performance, cost-effectiveness, and engineering applicability. Thanks to its vast internal specific surface area, activated carbon demonstrates outstanding equilibrium capacity and adsorption rates in the removal of most organic micropollutants and hydrophobic substances. In contrast, biochar—a product of biomass pyrolysis—typically has a lower specific surface area than activated carbon. Its abundance of surface functional groups and high cation exchange capacity confer unique chemical adsorption advantages for the adsorption of heavy metal ions and soil remediation. When modified, it can achieve adsorption capacities comparable to those of commercial activated carbon. Zeolites, owing to their regular crystal structure and excellent ion-sieving capacity, excel in the selective adsorption of ammonium ions, metal cations, and small-molecule gases such as CO_2_ and NH_3_. Their pore size limitations restrict their adsorption capacity for large-molecule organic compounds [192]. As an emerging class of porous materials, MOFs achieve extremely high specific surface areas and ultra-high adsorption capacities for specific gases (such as CO_2_ and VOCs) or aqueous pollutants through their designable topological structures. In particular, following functionalisation, they demonstrate the potential to outperform traditional materials [193].

In terms of estimated costs and economic viability, biochar demonstrates significant cost advantages, particularly when agricultural residues or municipal organic waste are used as precursors. Its production cost per unit mass is extremely low, and it can be produced locally, thereby substantially reducing logistics and processing costs. Although activated carbon has moderate raw material costs, its activation process is energy-intensive, and losses during regeneration increase its life-cycle costs. Its advantages in terms of engineering maturity and treatment capacity per unit bed volume mean it remains competitive in large-scale water treatment [194]. Zeolites, particularly natural zeolites, are inexpensive to source, and their ion-exchange regeneration costs are low, making them extremely cost-effective for the removal of inorganic ions [195]. The current synthesis costs of MOFs are high, primarily due to the price of organic ligands and complex synthesis conditions. Although the development of highly stable MOFs, such as Zr-based MOFs, and the optimization of solvent recovery processes in recent years are gradually reducing costs, the overall economic burden remains far higher than that of traditional adsorbents.

**Figure 12:**
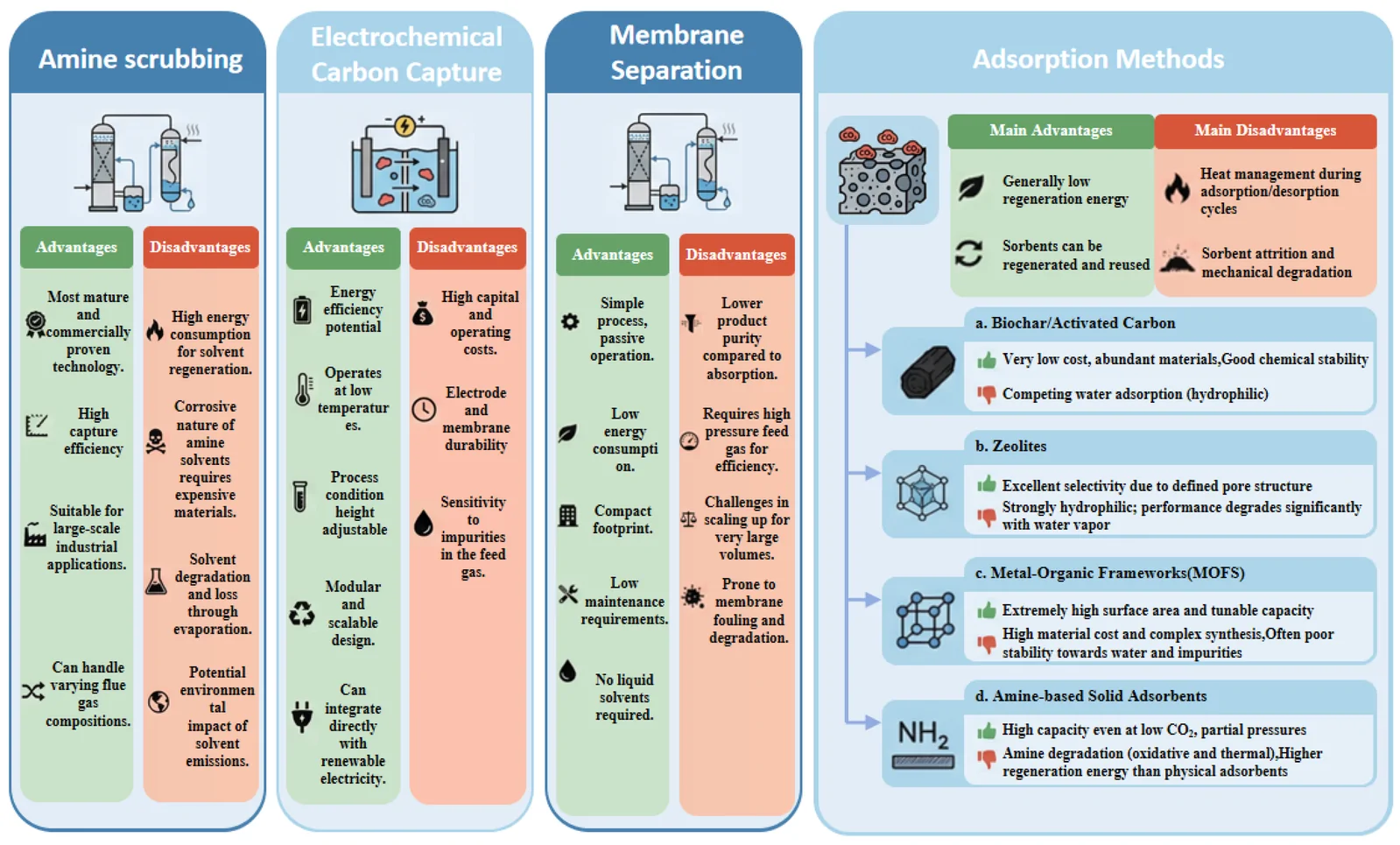
Pros and cons of CO_2_ adsorption tail gas treatment in industry.

Activated carbon and biochar typically operate stably at ambient to moderate temperatures. Activated carbon can undergo multiple cycles via high-temperature thermal regeneration or steam stripping. Each regeneration is accompanied by microporous collapse and irreversible loss of active sites. The regeneration of biochar typically involves low-temperature solvent elution or mild heat treatment. Its porous structure is more prone to degradation during repeated use, resulting in significant fluctuations in service life. Zeolites possess excellent thermal stability and are suitable for high-temperature desorption regeneration. Their crystal structure remains intact even after multiple ion-exchange or thermal cycles, ensuring a relatively long service life. MOFs are extremely sensitive to operating conditions. Most MOFs exhibit poor stability in aqueous phases or high-humidity environments. Although they can be regenerated through temperature variation or vacuum replacement, issues with hydrothermal stability limit their widespread engineering applications; only a few water-stable MOFs can achieve efficient cycling under precisely controlled operating conditions [196]. Figure 12 summarises the advantages and disadvantages of exhaust gas treatment using carbon dioxide adsorption in the industries mentioned above.

### 4.2. Adsorption Methods of SO_2_

Current technologies for removing SO_2_ from flue gas are primarily based on chemical reactions between sulfur dioxide and desulfurisation agents, and can broadly be divided into wet, dry, and semi-dry processes. In addition, catalytic oxidation and reduction technologies can also be used to remove sulfur dioxide.

Wet flue gas desulfurization (WFGD) is the most mature and widely applied technology, and the removal efficiency of SO_2_ is up to 90–98%. The process involves gas–liquid reactions between SO_2_ and alkaline slurries in spray or scrubber towers. SO_2_ dissolves into the liquid phase to form sulfurous acid, which is subsequently neutralized to form gypsum (CaSO_4_·2H_2_O) as a recoverable by-product [197]. Despite its high efficiency, WFGD systems are complex and capital-intensive. They also involve energy consumption, corrosion, visible plume formation, and wastewater generation, making them more suitable for large-scale, high-sulfur-emission sources.

Dry desulfurisation removes sulfur dioxide through gas–solid reactions without introducing liquid water. Powdered adsorbents (such as CaO or NaHCO_3_) are injected into the flue gas stream to react with the sulfur dioxide, forming a solid product which is subsequently captured by a particulate control device. This process is characterized by low capital costs, simple operation, and the absence of wastewater generation. However, achieving high desulfurisation efficiency typically requires the consumption of larger quantities of adsorbent, increasing operating costs. Consequently, dry systems are more suitable for low-sulfur applications or retrofit projects [198].

Semi-dry systems (such as spray-drying absorbers (SDA)) combine the characteristics of wet and dry methods. Atomized alkaline slurry is injected into high-temperature flue gas, where evaporation and reaction occur simultaneously to produce a dry solid product [199]. Such systems offer moderate removal efficiency (80–95%), simple equipment design, and do not generate wastewater discharge. However, their adsorbent consumption is typically higher than that of wet systems, resulting in higher operating costs.

Oxidation processes convert SO_2_ into SO_3_, which is then used to produce sulfuric acid. Reduction processes, on the other hand, aim to convert SO_2_ into elemental sulfur or H_2_S to enable resource recovery. Both methods can be applied in dry or wet systems and are increasingly being used for integrated pollution control, Table 2 summarized the characteristics for above methods and provided an economic analysis. 

Solid adsorbents such as activated carbon, zeolites, MOFs, and solid amines have shown significant potential for SO_2_ removal. Activated carbon provides a high surface area and tunable porosity, while metal oxide modification can further enhance adsorption and catalytic activity. Zeolites exhibit improved performance after ion exchange with transition metals. MOFs offer highly tunable structures and excellent adsorption capacity, although their stability remains a challenge [200]. Solid amine adsorbents exhibit high SO_2_ capacity and partial thermal reversibility, making them promising for deep desulfurization applications, the specific SO_2_ adsorption property for samples derived from various pre-treatment processes are summarized in Table 3.

### 4.3. Adsorption Methods of NOx

Current industrial technologies for NO*x* abatement mainly include selective catalytic reduction (SCR), selective non-catalytic reduction (SNCR), wet absorption, and adsorption processes. Each approach differs in reaction mechanism, operational conditions, and practical applicability.

SCR is the most widely applied and mature technology for large-scale NO*x* control [206]. In this process, ammonia (NH_3_) or urea is used as a reducing agent to convert NO*x* into harmless nitrogen (N_2_) and water (H_2_O) in the presence of a catalyst. SCR systems typically achieve high removal efficiencies (>90%) and are extensively deployed in industrial applications worldwide. The process operates within a relatively narrow temperature window (generally 300–400 °C) and relies on metal oxide catalysts, such as V_2_O_5_–WO_3_/TiO_2_, to facilitate the reaction. Despite its high efficiency, SCR technology faces several limitations. Catalyst deactivation may occur due to poisoning by SO_2_ and water vapor, while operational issues such as ammonia slip and the potential formation of secondary pollutants (e.g., N_2_O at elevated temperatures) remain significant concerns.

SNCR operates without a catalyst by injecting ammonia or urea directly into high-temperature flue gas, typically within a narrow temperature window of 850–1100 °C, where selective reduction of NO*x* occurs [207]. Compared with SCR, SNCR exhibits lower removal efficiencies, generally below 50% in practical applications. However, it offers substantially lower capital costs and simpler system design, making it an economically attractive solution for NO*x* control. A key limitation of SNCR is its narrow optimal temperature window, rendering it highly sensitive to fluctuations in flue gas temperature and thus challenging to control under variable operating conditions.

Wet absorption technologies remove NO*x* by dissolving it into liquid absorbents, followed by chemical reactions. However, NO, which accounts for approximately 90–95% of NO*x* in flue gas, has very low solubility in water. Therefore, pre-oxidation of NO to more soluble NO_2_ is typically required. Wet absorption processes can be categorized into several types: alkaline absorption, which primarily targets higher-valence NO*x* (e.g., NO_2_) and converts them into nitrite and nitrate species. Complexing absorbents, such as Fe^2+^–EDTA, can enhance NO solubility by forming stable nitrosyl complexes. However, these systems suffer from oxidative deactivation, and efficient regeneration remains a major challenge. Wet absorption technologies can simultaneously remove multiple pollutants, such as SO_2_ and NO*x*, and the equipment structures are relatively simple. Nevertheless, their efficiency is fundamentally limited by the low solubility of NO, necessitating the use of strong oxidants (e.g., H_2_O_2_, O_3_, NaClO_2_) to enhance performance. In addition, the generation of saline wastewater increases downstream treatment costs.

The desorption of NO*x* by zeolites occurs through a combination of physisorption (formation of nitrosyl complexes) and chemisorption involving interactions with framework cations. Adsorption technologies utilize porous solid adsorbents to remove NO*x* from flue gas via physical or chemical interactions. Once saturated, the adsorbents can be regenerated through temperature swing, pressure swing, or other desorption methods. Common adsorbents include: Activated carbon (AC), Zeolites, and MOFs. For AC, NO can be oxidized to NO_2_ on the carbon surface in the presence of O_2_, followed by the conversion of NO_2_ into nitrate species in the presence of moisture. The adsorption performance of NO*x* can be significantly enhanced by impregnation with metal oxides such as V_2_O_5_, CuO, and MNO_2_. The removal of NO*x* by zeolites is achieved through a combination of physisorption (formation of nitrosyl complexes) and chemisorption. Aminofunctionalised mesoporous silica can be used for the selective removal of SO_2_ from flue gas at low temperatures. As SO_2_ is more acidic than CO_2_, it binds preferentially to amine groups, making it suitable for the deep removal of low-concentration SO_2_ from flue gas. It offers better resistance to CO_2_ interference than ordinary inorganic materials. It possesses significantly greater corrosion resistance, making it suitable for long-term operation in sulfur-containing flue gas. However, the cost of raw materials for its large-scale production is high. MOFs possess high surface areas and tunable pore structures, offering excellent adsorption potential. However, their stability under realistic flue gas conditions remains insufficient, limiting large-scale industrial application. Adsorption-based methods offer advantages such as operational simplicity, flexibility, and the potential for NO*x* recovery. Under optimized conditions, relatively high removal efficiencies can be achieved. However, adsorption capacity strongly depends on the type and modification of the adsorbent. Moreover, competitive adsorption by coexisting gases, such as H_2_O and SO_2_, can significantly reduce adsorption performance. The regeneration efficiency and economic viability of adsorbents remain critical factors determining the practical feasibility of this technology. Table 4 summarized the characteristics for above methods and provided an economic analysis, the specific NOx adsorption property for samples derived from various pre-treatment processes are summarized in Table 5.

### 4.4. Adsorption Methods of VOCs

The treatment of VOCs emitted from waste incineration is a complex challenge that requires careful consideration of technical feasibility, economic costs, and environmental impacts. Existing VOCs abatement technologies can generally be classified into two categories: recovery technologies and destruction technologies [215]. Recovery methods aim to separate and recycle VOCs from exhaust streams, whereas destruction methods convert VOCs into harmless products such as CO_2_ and H_2_O through chemical or biological processes [215]. Common VOCs control technologies include adsorption, thermal and catalytic combustion, biodegradation, photocatalysis, membrane separation, and plasma-based processes [58].

Combustion-based methods are among the most widely used destruction technologies for VOCs removal, in which VOCs are oxidized into CO_2_ and H_2_O. These technologies include thermal oxidation, catalytic oxidation (CO), and regenerative thermal oxidation (RTO) [216]. RTO systems, in particular, achieve thermal recovery efficiencies exceeding 95% through ceramic heat storage media and are especially suitable for treating medium- to high-concentration VOCs streams. In catalytic combustion systems, catalysts such as Pd/Al_2_O_3_ have demonstrated complete methane oxidation at 600 °C, where interfacial oxygen vacancies significantly enhance catalytic activity. Recent advances in catalyst design have further improved the oxidation performance of VOCs. For instance, Ce-based catalysts have shown remarkable activity due to their redox properties and oxygen storage capacity. Ce-doped CuFe layered catalysts exhibit enhanced catalytic oxidation efficiency toward compounds such as N, N-dimethylacetamide and o-xylene. Moreover, metal oxide-modified Ce-based catalysts can achieve complete acetone oxidation at relatively low temperatures (≈195 °C) while simultaneously enabling synergistic NO*x* degradation [217]. Despite their high efficiency, combustion-based methods are energy-intensive and may produce secondary pollutants under suboptimal conditions, necessitating careful process optimization.

Biological treatment uses microbial metabolism to degrade VOCs and is considered an environmentally friendly, cost-effective approach. Typical systems include biofilters, biotrickling filters, and biological contact oxidation processes [218]. The performance of biological systems is strongly dependent on the biodegradability of VOCs. Refractory compounds such as halogenated hydrocarbons are difficult to degrade biologically, limiting the applicability of this method. To address these limitations, hybrid systems have been developed, such as the integration of biofiltration with UV-photocatalytic oxidation, which significantly enhances VOCs removal efficiency. In practical applications, a German-developed bioscrubbing process combining activated sludge and biofilm technologies has demonstrated stable removal efficiencies exceeding 90% for benzene-containing waste gases, highlighting the strong potential of biological approaches for specific VOC streams [219].

Membrane separation technology has shown significant potential for VOCs recovery and removal, as it selectively enriches and separates target compounds. Poly membranes are widely used owing to their excellent permeability and selectivity. To further enhance membrane performance, advanced porous materials such as covalent organic frameworks (COFs) and MOFs have been incorporated into polymer matrices to form mixed-matrix membranes (MMMs). COFs, with their tunable pore structures, high surface areas, and adjustable functionalities, are considered ideal fillers for membrane applications. Incorporation of COFs nanosheets into PEBA matrices enhances affinity toward aromatic compounds, improving both selectivity and permeability [220]. Similarly, MOF-based MMMs combine the high selectivity of MOFs with the mechanical robustness and processability of polymer matrices, offering improved VOCs separation performance [221].

Adsorption is one of the most widely applied technologies for VOCs control, particularly suitable for treating low-concentration and high-flow-rate exhaust streams. Common adsorbents include activated carbon, zeolites, and activated carbon fibers (ACFs). ACFs have attracted considerable attention due to their high specific surface area and rapid adsorption–desorption kinetics [222,223]. For example, ACFs can achieve a toluene adsorption capacity of up to 1.03 g/g while maintaining stable performance over 300 adsorption–desorption cycles. In addition to conventional materials, biochar derived from agricultural waste has emerged as a low-cost and sustainable adsorbent. For instance, Na_2_CO_3_-activated cattail-based biochar exhibits a dynamic adsorption capacity of up to 1.00 g/g for n-hexane, surpassing that of conventional activated carbon in terms of cost-effectiveness [58]. Adsorption-based technologies face challenges with adsorbent regeneration and reduced efficiency at high humidity, due to competitive adsorption with water vapor.

## 5. Cycle and Regeneration

Thermal regeneration is the most traditional and widely used method for restoring the adsorption performance of biochar; its core principle lies in desorbing pollutants at high temperatures and restoring the pore structure. However, conventional thermal regeneration is often associated with high energy consumption and irreversible damage to the biochar structure. Through molecular simulations combined with density functional theory, heteroatom-doped biochar can be rationally designed to optimize its surface electronic structure and adsorption energy barriers. This rational design not only enhances the selective capture capacity for specific pollutants such as CO_2_ but also reduces the activation energy during the regeneration process, enabling efficient regeneration at lower temperatures [224]. Furthermore, temperature control during the thermal regeneration process is crucial. As the temperature rises, lignin monomers undergo cracking and re-polymerisation, leading to increased aromatisation and graphitisation of the biochar. Moreover, the conjugated graphitic carbon network becomes more refined, significantly enhancing electrical conductivity, dielectric loss, and microwave absorption capacity. This facilitates the generation of reactive free radicals within the microwave system, enabling the degradation of organic pollutants. However, at excessively high temperatures, the proportion of C–O functional groups decreases, the (O+N)/C molar ratio falls, and surface polarity and hydrophilicity are weakened. This reduction in surface oxygen-containing functional groups consequently affects the subsequent adsorption performance of polar pollutants [225].

During the regeneration process, the application of specific pressure conditions can promote the desorption of adsorbates from the micropores. When treating macromolecular organic pollutants, pressure variations help to overcome diffusion limitations. Ultrasonic-assisted thermal regeneration technology combines the ultrasonic cavitation effect with thermal desorption. By creating a localized microenvironment of high pressure and high temperature, it accelerates the desorption and decomposition of pollutants, significantly improving regeneration efficiency. This synergistic effect not only lowers the overall regeneration temperature but also reduces thermal stress damage to the biochar matrix, extending the material’s cycle life [226]. The regeneration properties of biochar derived from different processes are illustrated in Table 6.

With regard to regeneration, for CO_2_ adsorption, biochar modified to increase its specific surface area typically exhibits virtually no decline in performance after 10 cycles. Biochar modified using amine-based chemistry shows relatively low regeneration capacity, as significant losses occur due to the loss of amines and the formation of urea by-products during regeneration cycles. Typically, the number of cycles for CO_2_-adsorbing biochar averages around 10, with a regeneration capacity of approximately 95%, physically activated biochar achieves higher capacities of 96–100%, while biochar modified with nitrogen-containing functional groups achieves lower capacities of 56–95%. However, in the case of amine-modified biochar adsorption, the adsorption of a small amount of moisture-containing CO_2_ enhances the cycle regeneration capacity [230]. This is primarily because, during the adsorption of dry CO_2_, there is no moisture shielding. CO_2_ occupies all sites in order of decreasing energy. However, CO_2_ at high energy levels binds more firmly to basic sites under N_2_ at 100 °C and is difficult to desorb completely. A small amount therefore remains in the ultraporous sites. This residue accumulates with each cycle, gradually reducing the adsorption capacity of the active sites. In contrast, when adsorbing humid CO_2_, H_2_O preferentially occupies highly polar, hydrophilic high-energy sites (oxygen-containing groups, pyrrole/pyridine nitrogen sites, and the narrowest pore mouths), protecting the sites that are most difficult to regenerate. However, if the humidity is too high, water blocks the micropores, significantly impairing adsorption and cycling performance.

SO_2_ is primarily chemically adsorbed onto carbon materials (forming H_2_SO_4_/sulphates), and their regeneration capacity after adsorption is relatively weak compared to that for other gases. Typically, carbon materials can retain 93% of their adsorption capacity after 6–8 cycles of SO_2_ adsorption; however, the retention rate after regeneration is lower when metal oxides are introduced. For example, Yavuz et al. used Fe_2_O_3_-modified biochar to adsorb SO_2_, whereby SO_2_ reacts with the carbon and Fe_2_O_3_ to form Fe_2_(SO_4_)_3_. During regeneration, if the temperature is too low, Fe_2_(SO_4_)_3_ does not decompose completely, leaving residual sulphates that block pores and coat active sites. If the temperature is too high, the iron oxides sinter and agglomerate, with some being reduced by the carbon to elemental Fe, while the pore structure of the carbon matrix collapses. Ultimately, this results in a reduction in effective active sites after regeneration, as well as the destruction and blockage of micropores, leading to a continuous decline in desulfurisation capacity throughout the adsorption–regeneration cycle.

There are fundamental differences in the adsorption mechanisms of NO and NO_2_ on the surface of biochar, resulting in markedly different regeneration strategies and decay behavior over time. The removal of NO_2_ by biochar relies primarily on chemical adsorption and surface dissociation reactions. Density functional theory (DFT) calculations indicate that, upon adsorption at active sites on biochar (such as potassium-doped sites or defect sites), NO_2_ molecules are highly prone to undergo a dissociation reaction to form nitrate (NO_3_^−^) and nitric oxide (NO), or to bind with surface oxygen- or nitrogen-containing functional groups to form stable surface complexes [157]. This chemical fixation mechanism means that the adsorption capacity for NO_2_ is generally much higher than that for NO, but it also results in a higher energy barrier that must be overcome during the regeneration process. In contrast, the physical adsorption of NO molecules on unmodified biochar is extremely weak, and effective chemical transformation is difficult to achieve under anaerobic conditions. To enhance the removal efficiency of NO, it is usually necessary to introduce transition metals (such as Mn, Fe and Cu) or alkali metals into the biochar, utilizing their catalytic oxidation to convert NO into NO_2_ or nitrate for storage [239]. This mechanism means that the regeneration of NO involves not only physical desorption but also the thermal decomposition or reduction in nitrates. Its regeneration behavior is thus closer to that of NO_2_, although the overall adsorption capacity remains limited by the oxidation efficiency [240].

The performance decline of biochar following the adsorption of VOCs is a complex physicochemical process, primarily attributable to the irreversible ‘residual heel’ effect caused by VOCs with high boiling points or strong interactions, as well as cumulative structural damage resulting from thermal aging during the regeneration process. These two mechanisms jointly determine the cycle life and long-term stability of biochar adsorbents. VOCs such as cyclohexane and toluene, which possess strong hydrophobicity and specific molecular sizes, readily penetrate deep into the micropores of biochar, where they form strong van der Waals forces or π–π electron donor–acceptor interactions. During conventional thermal or microwave regeneration processes, these molecules, deeply embedded within the micropores, are difficult to desorb completely, forming permanent or semi-permanent residual layers within the pores. Such residues not only occupy effective adsorption sites but also block the entrances to the pore channels, hindering the diffusion of subsequent adsorbate molecules. By contrast, although polar oxygen-containing VOCs such as acetone and ethanol form hydrogen bonds with the oxygen-containing functional groups on the biochar surface, their smaller molecular size and relatively low boiling points mean they are more readily desorbed at appropriate regeneration temperatures. Their adsorption retention rates are often higher than those of cyclohexane and toluene [241].

## 6. Summary and Outlook

### 6.1. Summary

As a sustainable adsorbent, biochar demonstrates significant potential for the removal of gaseous pollutants, including carbon dioxide, sulfur dioxide, nitrogen oxides and volatile organic compounds, due to its low cost, tunable physicochemical properties and excellent regenerative performance. The adsorption behavior of biochar is jointly influenced by the synergistic interaction between its pore structure, surface functional groups, and mineral composition. These characteristics can be effectively controlled through the selection of feedstock, pyrolysis conditions, and post-treatment strategies.

In fixed-bed adsorption systems, adsorbent particles are subjected to mechanical stresses arising from their own weight, the shear forces of the gas flow, and the pressure drop across the bed. Biochar is essentially a brittle, porous carbonaceous material; its framework consists of an interwoven network of aromatic microcrystalline and amorphous carbon, and lacks a dense cross-linked structure, resulting in relatively low compressive strength, wear resistance, and resistance to scouring. Under long-term operating conditions with high gas velocities, biochar particles are prone to attrition, fragmentation and even pulverization. This not only leads to a sharp rise in bed pressure drop, triggering channeling or localized fluidisation, but also results in adsorbent loss, thereby shortening the effective bed life. The characteristics of the feedstock and pyrolysis conditions significantly influence the mechanical strength of biochar. Feedstocks with higher lignin content, as well as biochar produced at higher pyrolysis temperatures, exhibit greater carbon framework density and a higher degree of microcrystallisation, and typically possess better mechanical integrity. However, whilst excessively high pyrolysis temperatures can increase micropore volume, they may reduce the structural toughness, making the material more prone to fragmentation. Furthermore, the ash content in biochar—particularly mineral components such as alkali metals, silicon and aluminum—often forms brittle inclusions and interfacial defects, which further weaken mechanical strength and generate large amounts of fine powder during abrasion. To address this issue, engineering approaches such as pelletisation processes or the addition of binders for carbonisation and densification are commonly employed. This requires striking a balance between enhancing mechanical strength and maintaining a high specific surface area and porosity.

With regard to the production of biochar, no breakthrough innovations have yet emerged in the field of biochar pre-treatment and modification. Most experimental studies published in recent years have employed similar modification methods using different feedstocks to produce biochar, whilst some have utilized novel materials to achieve higher adsorption capacities. However, the preparation processes are cumbersome, and production costs are high. The majority of these biochars exhibit adsorption capacities comparable to those of commercial activated carbon and have not yet achieved adsorption capacities significantly exceeding those of conventional solid adsorbents.

For industrial applications, although biochar has performed well in laboratory experiments, its industrial application still faces multiple structural barriers. Firstly, the heterogeneity of the raw materials leads to significant fluctuations in the quality of the biochar product. Biomass from different sources (such as wood, crop straw, and sewage sludge) has varying lignin, cellulose, and ash contents, which directly affect the pore structure and surface chemistry of the final biochar. This makes it difficult to establish uniform industrial standards and limits its large-scale application in precision flue gas treatment systems. Furthermore, in high-temperature flue gas environments, biochar that has reached adsorption saturation often faces the risk of structural collapse or deactivation of active sites, resulting in a limited number of reuses. Frequent replacement of the adsorbent not only increases operating costs but also creates new challenges regarding the disposal of solid waste, which runs counter to the original intent of the circular economy. Currently, the number of cycles for biochar adsorption of VOCs is typically five, and for acidic gases, ten. These figures reflect experimental design conventions rather than the upper limit of the material’s lifespan. The actual lifespan in engineered systems requires verification through long-term testing, typically involving 30–100 cycles of biochar.

As far as cost-effectiveness is concerned, the economic viability of biochar depends on production costs and the potential benefits derived from its adsorption performance and lifespan. Although biochar is inexpensive compared to commercial activated carbon, its pre-treatment and modification costs are relatively high. For instance, whilst the use of KOH to modify biochar—compared to other modifying agents—can significantly increase its specific surface area, its alkalinity can corrode plant equipment, leading to a substantial increase in operating costs. If, after modification, the biochar is rinsed with an acidic solution to neutralize it, this further increases costs. Whilst this is feasible under ideal laboratory conditions, large-scale biochar production in industrial settings is impractical, necessitating the search for alternative, more suitable modification materials. Regeneration performance determines whether a biochar adsorption system can be used in the long term and affects its economic viability. Regeneration efficiency varies significantly depending on the type of biochar and the modification method used. However, the biochar regeneration process is not entirely reversible. Typically, after five cycles, the adsorption capacity for pollutant gases begins to decline, and the rate of decline becomes particularly pronounced after ten cycles. Although thermal regeneration remains the most widely used method, it requires high temperatures for pyrolysis, resulting in significant energy consumption. Consequently, the challenge of preparing biochar without resorting to pyrolysis remains unresolved. The key economic breakthrough lies in extending the service life of biochar or developing high-value regeneration pathways. For example, if biochar that has reached adsorption saturation could be further utilized as a microbial carrier in anaerobic digestion systems to produce biogas, or reused as electrode material in supercapacitors, this would significantly enhance its life-cycle value.

From an environmental protection perspective, the introduction of metal- and metal oxide-modified biochar poses potential ecotoxicological risks. During adsorption operations and high-temperature regeneration, the loaded metals may be released into the environment as a result of wear and tear or chemical dissolution. In particular, under acidic, chlorine- or sulfur-containing wet flue gas conditions, changes in metal valence states and the formation of chlorides and sulphides may exacerbate the risk of metal ion leaching. The leakage of heavy metals such as lead, cadmium and chromium poses risks of acute and chronic toxicity, carcinogenicity and mutagenicity, as well as bioaccumulation. If not properly disposed of, discarded metal-modified biochar may be classified as hazardous waste. Essential measures to manage this risk include adopting strong complexation and immobilization strategies, selecting low-toxicity metals or non-metallic functional groups as substitutes, and establishing comprehensive closed-loop regeneration and disposal schemes.

### 6.2. Outlook

Future research should therefore focus on addressing these key challenges. Firstly, a systematic investigation of the competitive and synergistic adsorption mechanisms in multi-component gas systems is crucial for understanding adsorption behavior in real-world environments. Secondly, priority should be given to the rational design of advanced functionalized biochar materials with tailored pore structures and surface chemical properties, in order to enhance their selectivity and adsorption capacity for target pollutants. Thirdly, biochar production and activation processes need to be optimized to achieve an optimal balance between adsorption performance, economic viability, and environmental sustainability. The key economic breakthrough lies in extending the service life of biochar or developing high-value regeneration pathways. For example, if biochar that has reached adsorption saturation could be further utilized as a microbial carrier in anaerobic digestion systems to produce biogas, or reused as electrode material in supercapacitors, this would significantly enhance its life-cycle value. Finally, comprehensive techno-economic analyses and life-cycle assessments (LCA) should be conducted to evaluate the feasibility of large-scale application of biochar-based adsorption systems. These efforts will provide key insights into process optimization, cost-effectiveness and environmental impact, enhancing the ability of biochar to adsorb pollutants in practical industrial applications.

## Figures and Tables

**Table 1:** Methods for the preparation of biochar-based materials and their carbon dioxide adsorption properties at atmospheric pressure.

Biomass Feedstock	Biochar Modification Method	BET Surface Area (m^2^/g)	Adsorption Temperature (°C)	Adsorption Pressure (bar)	Concentration	Gas Flow Rate (mL/min)	Relative Humidity	CO_2_ Adsorption Capacity (mg/g)	Adsorption Capacity Per Surface area (mg/m^2^)	Ref.
Rice Husk	NH_3_ treatment at 700 °C for 30 min	451.02	30	1	100%	100	Dry	77.9	0.172	[186]
Shaddock Peel	Impregnation with ZnCl_2_; pre-carbonization at 400 °C	1376	0	1	100%	Static volumetric method	Dry	212.52	0.154	[187]
Distiller’s Grains	KOH modification	1059	0	1	100%	100	Dry	279	0.263	[188]
70% Chicken Manure + 30% Chicken Droppings	KOH impregnation	1281.6	25	1	100%	100	Dry	128.5	0.100	[188]
Food Waste + Wood Waste	KOH modification	841.3	25	1	100%	Static volumetric method	Dry	141.97	0.168	[143]
Seaweed	Microwave pyrolysis; activation with Na_2_S_2_O_3_·5H_2_O + KOH alkalization	2890	0	1	100%	100	Dry	186.12	0.064	[128]
Bamboo	Co-pyrolysis with ZIF-8	989.3	30	1	100%	100	Dry	107	0.108	[137]
Alkaline Lignin	Acid washing (10% acid solution, 30 min) + ultrasonic treatment (100 W, 5 min); pyrolysis	1134	0	1	100%	50	Dry	178.75	0.157	[189]
Bagasse and hickory wood	Slow pyrolysis of bagasse and hickory wood (600 °C)	-	25	1	100%	-	-	73.55	-	[190]
Rice straw	Microwave pyrolysis of rice straw (200 W, maximum temperature ~300 °C)	-	20	-	100%	-	-	80	-	[191]

**Table 2:** Summary of common desulfurization technologies.

Technology Type	Typical Desulfurization Efficiency	Reaction Medium	By-Product	Energy Consumption	Capital Cost	Operational Cost (OpEx)	Key Application Scenarios
Wet Desulfurization	90–98%	Gas–liquid (alkaline solution)	Gypsum/Sludge	High	High	Low	Large coal-fired power plants, high-flow and long-life scenarios
Dry Desulfurization	Up to 90%	Gas–solid (powder absorbent)	Dry powder	Generally low (high absorbent demand)	High	High	Low-sulfur coal, old plant retrofits, short-life scenarios
Semi-dry Desulfurization	80–95%	Gas–liquid (spray drying)	Dry powder	Low	Low	High	Power plants, cement plants, waste incineration plants
Oxidation/Reduction Method	Dependent on process	Gas–liquid (oxidant/reductant)	Sulfuric acid/Elemental sulfur	Medium	High	High	Sulfur resource recovery, chemical industry

**Table 3:** Methods for the preparation of biochar-based materials and their SO_2_ adsorption properties at atmospheric pressure.

Biomass Feedstock	Biochar Modification Method	BET Surface Area (m^2^/g)	Adsorption Temperature (°C)	Adsorption Pressure (bar)	Concentration	Gas Flow Rate (mL/min)	Relative Humidity	SO_2_ Adsorption Capacity (mg/g)	Adsorption Capacity Per Surface Area (mg/m^2^)	Ref.
Coconut Shell	Two-step pyrolysis; cetyltrimethylammonium bromide (CTAB) modification; CO_2_ activation	1180.6	30	1	-	-	Dry	29.44	0.025	[201]
Coconut Shell	Impregnation with Cu(NO_3_)_2_ solution; steam activation	843	25	1	400 ppm SO_2_ + 7.68 vol% O_2_	-	Dry	35	0.042	[27]
Bamboo	Two-step pyrolysis with ZnIF-8; metal framework-assisted activation	308.6	30	1	1000 ppm	100	Dry	166	0.538	[202]
Oil Palm Stem	Two-step pyrolysis; CO_2_ activation	270.6	100	1	300 ppm	300	Dry	73.89	0.273	[203]
Corn Cob	MDEA impregnation; CO_2_ activation	654	120	1	2000 ppm	100	Dry	216.19	0.331	[204]
Soybean Straw	NH_3_ modification	371.11	30	1	10,000 ppm	100 (N_2_ 90 mL/min + SO_2_ 10 mL/min)	Dry	175.9	0.474	[144]
Rice Straw	MgO loading; MgCl_2_·6H_2_O as Mg source; TEPA as precipitant	139.2	25	1	3000 ppm	400	Dry	260	1.868	[205]
Birch Sawdust	Steam activation	590	20	1	50 ppm	30	Moist	76.9	0.130	[145]
Shengli Lignite	CO_2_ and SO_2_ co-activation	342.03	75	1	1700 ppm, 6% O_2_, 8% H_2_O	500	8 vol% H_2_O	89.26	0.261	[149]
Olive Stone	H_3_PO_4_ chemical activation	1456.3	30	1	5000 ppm	-	Dry	380.1	0.261	[50]

**Table 4:** Summary of common NO*x* removal technologies.

Technology Type	Typical NO*x* Removal Efficiency	Temperature Window	Key Operation Parameters	Technical Maturity & Main Limitations
SCR	Up to 90%	300–400 °C	Catalyst type, dosage	Mature, high efficiency; high catalyst cost, regular replacement required
SNCR	Slightly below 50%	850–1100 °C	Reductant dosage, injection position	Low cost, simple operation; low efficiency, temperature-sensitive, ammonia escape risk
Wet Absorption	70–96%	Room temperature to medium temperature	Oxidation step (variable)	Produces wastewater, high chemical consumption and wastewater treatment cost
Adsorption Technology	62–95%	Room temperature to medium temperature	Adsorbent type, regeneration frequency	Realizes NO*x* resource utilization; limited adsorption capacity, sensitive to H_2_O/SO_2_, high regeneration cost

**Table 5:** Methods for the preparation of biochar-based materials and their NO*x* adsorption properties at atmospheric pressure.

Biochar	Adsorbed Gas	BET Surface Area (m^2^/g)	Adsorption Temperature (°C)	Adsorption Pressure (bar)	Concentration	Gas Flow Rate (mL/min)	Relative Humidity	Adsorption Capacity (mg/g)	Ref.
Cannabis sativa straw with 30 wt% modified laterite	NO_2_	-	Room temperature	1	500 ppb	-	Dry	14.65	[152]
Supercritical CO_2_ extraction residue of nettle seeds, physically activated	NO_2_	1024	Room temperature	1	1000 ppm	450 (NO_2_:20%; Air:80%)	70%RH	59.1	[208]
Extraction residue of chaga mushroom	NO_2_	743	Room temperature	1	1000 ppm	450	70%RH	40.9	[209]
Supercritical extraction residue of hops	NO_2_	-	Room temperature	1	1000 ppm	450	70%RH	79.2	[210]
Wood shavings for animal bedding	NO_2_	203	250	1	511 ppm	833	Dry	43.54	[151]
Olive stone with physically activated by steam at 750 °C	NO_2_	-	Room temperature	1	1000 ppm	450	70RH	131	[211]
Rubber seed shell with KOH	NO	-	30	1	200 ppm	100	Dry	470.5	[212]
Oak wood chips by CO_2_ activate	NO	723	30	1	2.5–7.5 ppmv	1000	Dry	Removal rate: 99.9%	[213]
Rice husk biochar pyrolyzed at 450 °C	NO	3.9	Room temperature	1	90 ppm	1200	Dry	95.7	[214]
Sunflower seed shell biochar pyrolyzed at 450 °C	NO	3.9	Room temperature	1	90 ppm	1200	Dry	31.6	[214]

**Table 6:** Number of pollutant gases adsorption cycles and regeneration capacity of biochar-based materials.

Biochar Production	Adsorbed Gas	Initial Capacity	Cycle Number	Retention Rate After Regeneration	Ref.
Straw and woody biochar	CO_2_	41.2–45.9 mg/g	10	96.57–98.94%	[227]
Lignin-impregnated biochar (microwave-assisted)	CO_2_	134.46 mg/g	10	89.79–99.06%	[228]
Herb residue biochar pellets (K_2_CO_3_/K_2_FeO_4_)	CO_2_	2.20 mmol/g (60 °C, 20% CO_2_ by weight)	>9	99.1%	[229]
N-doped biochar	CO_2_	3.81 mmol/g (25 °C)	10	88% (dry)/95% (30% RH)	[230]
Photo-oxidation modified rice straw biochar	CO_2_	3.5 mmol/g (12% CO_2_, 25 °C)	10	97.1%	[231]
Ultrasonic-assisted alkali-activated biochar	CO_2_	2.79 mmol/g (70 °C)	15	56%	[232]
Lignin-impregnated microporous biochar	CO_2_	77.0–102.9 mg/g	10	93.98–98.21%	[233]
Steam-activated wood-derived biochar	SO_2_	590 m^2^/g	6	Higher	[145]
ZIF-8 assisted pyrolysis bamboo biochar (N-doped)	SO_2_	166 mg/g	8	93%	[202]
Iron oxide modified biochar (microwave-assisted preparation)	SO_2_	146.31 mg/g	5	32%	[234]
Petroleum coke-based activated carbon (KOH/NaOH/K_2_CO_3_-KOH)	SO_2_	0.064–0.148 mmol/g (moist)	10	No change	[147]
Monolithic Co_3_O_4_/activated carbon	SO_2_	123.1 mg/g	2	94.4%	[235]
Ball-milled biochar (hickory wood, 450/600 °C)	VOCs	103.4 mg/g (acetone: highest)	5	Cyclohexane 81.2%, toluene 90.5%, acetone 91.4%	[161]
Ball-milled corn stover biochar modified with H_2_O_2_/NH_4_OH	VOCs	50–100 mg/g	5	Type O: 88.0–92.2%; Type N: 92.2–95.4%	[26]
Wheat straw/sugarcane bagasse biochar	VOCs	108.0–130.2 mg/g	5	88–96%	[160]
Nitrogen-doped porous corn cob biochar	Toluene	603 mg/g	4	95%	[236]
Duckweed-based porous carbon activated by KOH	Toluene	910.97 mg/g	4	90%	[237]
Chemically activated hydrothermal carbon	Acetone/Cyclohexane	50.6–159.7 mg/g	5	92–94%	[238]
Lignin-impregnated microporous biochar	Benzene/Acetone	31.4–81.0 mg/g	10	93.98–98.21%	[233]

## Data Availability

No new data were created or analyzed in this study. Data sharing is not applicable to this article.

## Abbreviations

The following abbreviations are used in this manuscript:

VOCs	Volatile Organic Compounds
MOFs	Metal–Organic Frameworks
pH_p_zc	Zero-Charge Point
DEA	Diethylamine
EDC	1-Ethyl-3-(3-Dimethylaminopropyl) Carbodiimide
XPS	X-Ray Photoelectron Spectroscopy
DFT	Density Functional Theory
WFGD	Wet Flue Gas Desulfurization
ACFs	Activated Carbon Fibers
LCA	Life-Cycle Assessments
